# Structural and mechanistic insights into the CRISPR inhibition of AcrIF7

**DOI:** 10.1093/nar/gkaa690

**Published:** 2020-08-18

**Authors:** Iktae Kim, Jasung Koo, So Young An, Suji Hong, Donghyun Ka, Eun-Hee Kim, Euiyoung Bae, Jeong-Yong Suh

**Affiliations:** Department of Agricultural Biotechnology, Seoul National University, Seoul 08826, Korea; Department of Agricultural Biotechnology, Seoul National University, Seoul 08826, Korea; Department of Agricultural Biotechnology, Seoul National University, Seoul 08826, Korea; Department of Agricultural Biotechnology, Seoul National University, Seoul 08826, Korea; Department of Agricultural Biotechnology, Seoul National University, Seoul 08826, Korea; Protein Structure Research Team, Korea Basic Science Institute, Ochang 28119, Korea; Department of Agricultural Biotechnology, Seoul National University, Seoul 08826, Korea; Research Institute of Agriculture and Life Sciences, Seoul National University, Seoul 08826, Korea; Department of Agricultural Biotechnology, Seoul National University, Seoul 08826, Korea; Research Institute of Agriculture and Life Sciences, Seoul National University, Seoul 08826, Korea

## Abstract

The CRISPR–Cas system provides adaptive immunity for bacteria and archaea to combat invading phages and plasmids. Phages evolved anti-CRISPR (Acr) proteins to neutralize the host CRISPR–Cas immune system as a counter-defense mechanism. AcrIF7 in *Pseudomonas aeruginosa* prophages strongly inhibits the type I-F CRISPR–Cas system. Here, we determined the solution structure of AcrIF7 and identified its target, Cas8f of the Csy complex. AcrIF7 adopts a novel β1β2α1α2β3 fold and interacts with the target DNA binding site of Cas8f. Notably, AcrIF7 competes with AcrIF2 for the same binding interface on Cas8f without common structural motifs. AcrIF7 binding to Cas8f is driven mainly by electrostatic interactions that require position-specific surface charges. Our findings suggest that Acrs of divergent origin may have acquired specificity to a common target through convergent evolution of their surface charge configurations.

## INTRODUCTION

Bacteria and archaea employ diverse defense mechanisms to fend off invading bacteriophages and foreign plasmids (1). Among others, the clustered regularly interspaced short palindromic repeat (CRISPR) and CRISPR-associated (Cas) proteins constitute an RNA-guided adaptive immune system to search and destroy invading genomes (2,3). Cas proteins first assemble into an integrase complex that cleaves and inserts invading genomic sequences into CRISPR loci of host genomes. Acquired DNA sequences are then transcribed and processed into mature guide RNAs. Finally, guide RNAs assemble with Cas proteins to form interference complexes that effectively detect and destroy foreign nucleic acids complementary to the guide RNA sequences.

**Figure 1.**
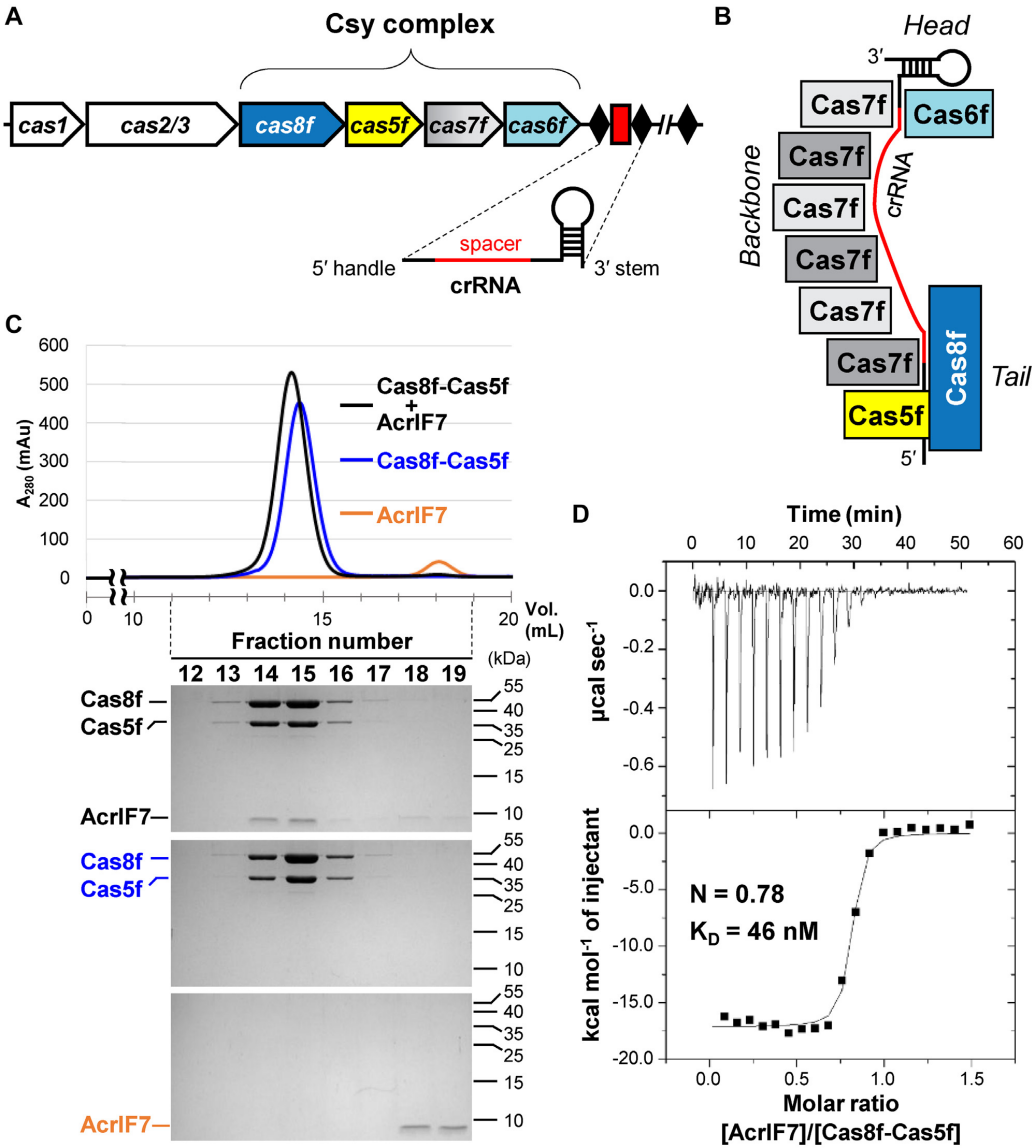
AcrIF7 interacts tightly with the Cas8f–Cas5f heterodimer. (**A**) Schematic representation of the type I-F CRISPR–Cas locus. The type I-F CRISPR–Cas system contains six *cas* genes, four of which encode the Cas proteins that constitute the Csy complex. The CRISPR array is comprised of invariable repeats (*black* diamonds) interspaced with variable phage-derived spacer sequences (*red* rectangle). (**B**) The architecture of the Csy complex. The crRNA-guided surveillance complex displays a subunit stoichiometry of Cas8f_1_:Cas5f_1_:Cas7f_6_:Cas6f_1_:crRNA_1_. (**C**) Analytical SEC experiments for the interactions between AcrIF7 and the Cas8f–Cas5f heterodimer. AcrIF7 (20 μM) co-eluted with Cas8f–Cas5f heterodimer (20 μM). Elution fractions were analyzed by SDS-PAGE. Uncropped gel images are shown in Supplementary Figure S9. (**D**) ITC trace for the binding of AcrIF7 to the Cas8f–Cas5f heterodimer. Experimentally determined stoichiometry (*N*) and equilibrium dissociation constant (*K*_D_) values are indicated.

The CRISPR–Cas system consists of two classes according to the composition of the interference complex: Class 1 employs a multi-subunit protein complex, whereas Class 2 employs a single effector protein for target interference (4,5). The Class 1 CRISPR–Cas system is further divided into 3 types (types I, III and IV) and 12 subtypes according to the participating Cas proteins and their targeting nucleic acids. The type I-F CRISPR–Cas system employs four Cas proteins (Cas5f–8f; also known as Csy2, Csy4, Csy3 and Csy1, respectively) and CRISPR RNA (crRNA) to form a multi-subunit complex for target DNA recognition (Figure 1A). The crRNA-guided surveillance complex (Csy complex) features a heterodimeric Cas8f–Cas5f ‘tail’ subunit that binds to the 5' handle region of crRNA, six Cas7f subunits that form a spiral ‘backbone’ encompassing the crRNA spacer region, and a Cas6f ‘head’ subunit that processes the 3′ stem–loop repeat region of crRNA (Figure 1B). Once the Csy complex binds to the matching DNA sequence, it recruits the Cas2/3 helicase-nuclease for processive degradation of the DNA target.

Bacteriophages have evolved anti-defense proteins to neutralize the host CRISPR–Cas system, and various anti-CRISPR (Acr) proteins have been found in phages and mobile genetic elements (6). To date, more than ten Acr proteins have been reported to inhibit the type I-F CRISPR–Cas system (7–9). Previously characterized type I-F Acrs either directly bind to the Csy complex to block target DNA binding or prevent the recruitment of Cas3 nuclease to the Csy complex (Supplementary Table S1) (10–13).

AcrIF7 was discovered from prophages of *Pseudomonas aeruginosa* via the ‘guilt-by-association’ bioinformatics approach (8). AcrIF7 effectively rescues CRISPR-sensitive phages in *P. aeruginosa* strains with type I-F CRISPR–Cas activity (8), but its inhibitory mechanism remains unknown. Here, we determined the solution structure of AcrIF7 using NMR spectroscopy, demonstrating that AcrIF7 adopts a novel α/β fold with dense negative surface charges. AcrIF7 targets the Cas8f subunit of the Csy complex and competes for the same binding interface with AcrIF2. Extensive mutagenic analyses revealed that AcrIF7 associated with the highly conserved dsDNA binding site of Cas8f, primarily via electrostatic interactions. Our study provides structural and mechanistic insights into the function of AcrIF7, expanding the knowledge of Acr inhibitors against type I-F CRISPR immunity.

## MATERIALS AND METHODS

### Cloning, expression and purification

The synthetic gene of AcrIF7 was cloned into pET28a with an N-terminal (His)_6_-maltose binding protein (MBP) tag and a tobacco etch virus (TEV) protease cleavage site. The mutant AcrIF7 genes were generated using polymerase chain reaction (PCR) with mutagenic primers (Supplementary Table S2). The wild type (WT) and mutant constructs were transformed into *Escherichia coli* BL21(DE3) cells, and the cells were cultured in LB medium at 37°C until the optical density at 600 nm reached 0.6. Protein expression was induced by the addition of 0.5 mM isopropyl β-d-1-thiogalactopyranoside followed by incubation at 17°C for 16 h. The *E. coli* cells were harvested by centrifugation and resuspended in the lysis buffer (20 mM 4-(2-hydroxyethyl)-1-piperazineethanesulfonic acid (HEPES), pH 7.0, 300 mM NaCl, 5 mM β-mercaptoethanol (BME) and 10% (w/v) glycerol). After sonication and centrifugation, the supernatant was loaded onto a 5-ml HisTrap HP column (GE Healthcare) pre-equilibrated with the binding buffer (20 mM HEPES, pH 7.0, 300 mM NaCl, 5 mM BME, 10% (w/v) glycerol and 30 mM imidazole). The column was washed with the same buffer, and a linear gradient of imidazole (up to 450 mM) was applied to elute the bound protein. The N-terminal (His)_6_-MBP tag was cleaved by TEV protease and separated with the HisTrap HP column (GE Healthcare). Proteins were further purified by size-exclusion chromatography (SEC) using a HiLoad 16/60 Superdex 75 column (GE Healthcare) equilibrated with buffer (20 mM HEPES, pH 7.0, 150 mM NaCl and 2 mM 1,4-dithiothreitol (DTT)).

The synthetic genes of Cas8f and Cas5f from *Xanthomonas albilineans* were cloned into pET28a with an N-terminal (His)_6_-MBP tag and a TEV protease cleavage site, and pET21a without a tag, respectively. The mutant Cas8f genes were generated by site-directed mutagenesis using mutagenic PCR primers. (His)_6_-MBP-Cas8f and untagged Cas5f were co-expressed in *E. coli* BL21(DE3) cells transformed with both Cas8f and Cas5f constructs. Proteins were expressed similar to AcrIF7 as described above. The (His)_6_-MBP-tagged Cas8f–Cas5f heterodimer was loaded onto a 5-ml HisTrap HP column (GE Healthcare) pre-equilibrated with the binding buffer (20 mM tris(hydroxymethyl)aminomethane (Tris)–HCl, pH 7.5, 300 mM NaCl, 5 mM BME, 10% (w/v) glycerol and 30 mM imidazole). After washing the column with the same buffer, we eluted the Cas8f–Cas5f heterodimer by applying a linear gradient of imidazole (up to 450 mM). The (His)_6_-MBP tag on the Cas8f protein was cleaved by TEV protease and separated with a HisTrap HP column. Contaminating proteins were further removed by anion exchange chromatography. The sample was loaded onto a Mono Q 5/50 GL column (GE Healthcare) pre-equilibrated with buffer (20 mM 1,3-bis(tris(hydroxymethyl)methylamino)propane, pH 8.5, 150 mM NaCl and 5 mM BME), and the unbound Cas8f–Cas5f heterodimer was pooled. Finally, the Cas8f–Cas5f heterodimer was purified by SEC using a HiLoad 16/60 Superdex 200 column (GE Healthcare) equilibrated with buffer (20 mM Tris–HCl, pH 7.5, 150 mM NaCl, 2 mM DTT and 5% (w/v) glycerol).

The synthetic gene of AcrIF2 was cloned into pET32a containing an N-terminal thioredoxin-(His)_6_ tag and a TEV protease cleavage site. The protein was expressed and purified in the same manner as described for AcrIF7, except for the use of 20 mM sodium phosphate buffer instead of the HEPES buffer. AcrIF2 was finally purified by SEC using a HiLoad 16/60 Superdex 75 column (GE Healthcare) equilibrated with buffer (20 mM sodium phosphate, pH 7, 150 mM NaCl, 2 mM DTT and 5% (w/v) glycerol).

### Analytical SEC

Analytical SEC was performed using a Superdex 200 10/300 GL column (GE Healthcare) equilibrated with buffer (20 mM Tris–HCl, pH 7.5, 150 mM NaCl and 2 mM DTT). Proteins (20 μM each) were mixed and incubated in 700 μl buffer at 4°C for 1 h, and loaded onto the column at a flow rate of 0.5 ml/min. Elution fractions were analyzed by sodium dodecyl sulfate–polyacrylamide gel electrophoresis (SDS-PAGE) and visualized by the Coomassie staining.

### Isothermal titration calorimetry (ITC)

The equilibrium dissociation constants between AcrIF7 (or its mutants) and Cas8f–Cas5f (or its mutants) were measured in buffer (20 mM Tris–HCl, pH 7.5, 150 mM NaCl and 1 mM tris(2-carboxyethyl)phosphine (TCEP)) at 25°C using an iTC_200_ Calorimeter (Malvern). 35 μM Cas8f–Cas5f was placed in the cell and titrated with 250 μM AcrIF7 in the syringe. Nineteen 2-μl aliquots of proteins were titrated into the cell. ITC data were analyzed using the Origin software provided with the instrument.

### Light scattering

Static light scattering data were obtained using a Superdex 200 Increase 10/300 GL column (GE Healthcare) coupled with a DAWN HELEOS II (18-angle) light scattering detector (Wyatt Technology) and an Optilab T-rEX refractive index detector (Wyatt Technology). The column was equilibrated with buffer (20 mM HEPES, pH 7.0, 150 mM NaCl and 2 mM DTT,). 100 μl of AcrIF7 (23.2 mg/ml) was loaded onto the column at a flow rate of 0.5 ml/min at 25°C. Data were analyzed using the ASTRA 6 software (Wyatt Technology).

### NMR spectroscopy

To produce ^13^C,^15^N-labeled AcrIF7 for NMR spectroscopy, *E. coli* BL21(DE3) cells containing the AcrIF7 construct were cultured in minimal medium supplemented with ^15^NH_4_ and ^13^C_6_-glucose as sole nitrogen and carbon sources, respectively, at 37°C until the optical density at 600 nm reached 0.8. The labeled protein was expressed and purified as described above for unlabeled AcrIF7. The NMR sample was prepared as 0.6 mM ^13^C,^15^N-AcrIF7 in buffer (20 mM HEPES, pH 7.0, 150 mM NaCl, 2 mM DTT and 10% (v/v) D_2_O). NMR spectra were collected at 25°C on Bruker AVANCE III 600, 700, 800 and 900 MHz spectrometers equipped with a *z*-shielded gradient triple resonance cryoprobe. NMR spectra were processed using the NMRPipe program (14) and analyzed using the PIPP/CAPP/STAPP (15), NMRView (16) and NMRFAM-SPARKY (17) programs. Sequential assignment was performed using 3D triple resonance through-bond scalar correlation experiments including HNCO, HN(CA)CO, HNCA, HN(CO)CA, HNCACB and CBCA(CO)NH experiments. Side chain assignment was performed using HBHA(CO)NH, ^15^N-seperated TOCSY, and HCCH-TOCSY experiments. ^13^C-seperated NOESY and ^15^N-seperated NOESY experiments were obtained using a mixing time of 120 ms. Residual ^1^*D*_NH_ dipolar couplings were obtained by taking the difference in the ^1^*J*_NH_ splitting values measured in aligned (11.5 mg/ml of pf1 phage; ASLA Biotech) and isotropic media using 2D in-phase/antiphase ^1^H–^15^N HSQC spectra. {^1^H}–^15^N heteronuclear NOE measurements were acquired using 3 s of 120° ^1^H pulses separated by 5 ms intervals using a previously employed pulse program (18). For NMR titration, ^1^H–^15^N HSQC spectra were recorded for 0.1 mM ^15^N-AcrIF7 titrating with 0.01–0.13 mM Cas8f–Cas5f in buffer (20 mM Tris–HCl, pH 7.5, 150 mM NaCl and 1 mM TCEP) at 25°C.

### Structure calculation

Interproton distance restraints were derived from the NOE spectra and classified into distance ranges according to the peak intensity. ϕ/ψ torsion angle restraints were derived from backbone chemical shifts using the program TALOS+ (19). Structures were calculated by simulated annealing in torsion angle space using the Xplor-NIH program (20). The target function for simulated annealing included covalent geometry, a quadratic van der Waals repulsion potential, square-well potentials for interproton distance and torsion angle restraints, hydrogen bonding, harmonic potentials for ^13^Cα/^13^Cβ chemical shift restraints (21), and a multidimensional torsion angle database potential of mean force (22). Structures were displayed using the PyMOL software (The PyMOL Molecular Graphics System, Version 2.0 Schrödinger, LLC.).

### Circular dichroism (CD) spectroscopy

CD spectra were obtained for protein samples in 500 μl buffer (10 mM sodium phosphate, pH 7.2) at 25°C using a J-815 circular dichroism spectropolarimeter (Jasco).

### Molecular Docking

The model of the AcrIF7:Cas8f complex was generated using the HADDOCK 2.4 web version with CNS (23). We used the structural coordinates of AcrIF7 (this study) and *P. aeruginosa* Cas8f (PDB code 5UZ9 and chain A), and employed key residues at the interface identified by SEC and ITC as ambiguous interaction restraints. Active residues crucial for the interaction were defined as follows: Asp13, Glu33 and Glu34 for AcrIF7; Lys28 and Lys247 for *P. aeruginosa* Cas8f. These residues significantly reduced the binding affinity in the ITC experiments upon charge-inversion mutations. Passive residues were automatically defined as those within 6.5 Å around the active residues. One thousand structures were generated via docking with rigid body energy minimization from random initial states, and 200 lowest energy structures were selected for subsequent semi-flexible simulated annealing and an explicit water refinement. The structure with the best HADDOCK score was displayed using the PyMOL software (The PyMOL Molecular Graphics System, Version 2.0 Schrödinger, LLC.).

## RESULTS

### AcrIF7 targets the Cas8f–Cas5f tail of the Csy complex

Acr proteins against type I-F CRISPR–Cas systems have diverse amino acid sequences and inhibition mechanisms (Supplementary Table S1). AcrIF2, AcrIF6 and AcrIF10 that interact with the heterodimeric Cas8f–Cas5f tail of the Csy complex (Figure 1B) compete with DNA for a crucial binding site, suggesting their roles as DNA mimics (10–12). Consistent with these observations, all three Acr proteins are highly acidic with low (<4.0) theoretical isoelectric point (p*I*) values (Supplementary Table S1). Interestingly, AcrIF7, whose mechanism remains unknown, also has a low (∼3.9) p*I* value (Supplementary Table S1). This suggests AcrIF7 may also bind to the Cas8f–Cas5f subunit of the Csy complex to inhibit type I-F CRISPR–Cas activity.

Using individually purified recombinant proteins, we asked whether AcrIF7 interacts with the *X. albilineans* Cas8f–Cas5f heterodimer. We previously showed that *X. albilineans* Cas8f and Cas5f proteins stably form a heterodimeric complex that is capable of binding the 5′ crRNA handle (24). AcrIF2, a previously characterized type I-F Acr protein, associates tightly with the *X. albilineans* Cas8f–Cas5f heterodimer with an equilibrium dissociation constant (*K*_D_) of 7.2 nM (24). AcrIF2 also shows similar affinity for the complete *P. aeruginosa* Csy complex (10,11). These observations confirm the biological relevance of the *X. albilineans* Cas8f–Cas5f subunit in testing interactions with Acr proteins. In analytical SEC experiments, we found AcrIF7 co-eluted with the *X. albilineans* Cas8f–Cas5f heterodimer (Figure 1C), indicating that AcrIF7 binds the ‘tail’ region of the Csy complex. In an ITC experiment, we observed a 1:1 binding stoichiometry between AcrIF7 and the Cas8f–Cas5f subunit with a *K*_D_ value of 46 ± 14 nM (Figure 1D). The interaction of AcrIF7 and Cas8f–Cas5f was driven by favorable enthalpic contribution that outweighed unfavorable entropic contribution, which generally indicates hydrogen bonds and electrostatic interactions at the interface (Table 1). Notably, the relative enthalpic and entropic contributions were similar in the interaction between AcrIF2 and Cas8f–Cas5f, suggesting a common driving force in both interactions (Table 1). We further examined the ^1^H–^15^N HSQC spectra of ^15^N-labeled AcrIF7 while titrating the Cas8f–Cas5f complex in an effort to identify the binding interface (Supplementary Figure S1A). We observed a gradual line-broadening of backbone and side-chain amide resonances without any noticeable chemical shift changes. The amide resonances of free and bound AcrIF7 are thus likely in slow exchange on the chemical shift time scale. The large size of the resulting complex of AcrIF7 with Cas8f–Cas5f (87.6 kDa) led to a broadening of the AcrIF7 resonances. We then examined whether a comparison of normalized intensity ratios between AcrIF7 resonances could locate potential binding interfaces. We observed, however, only modest variations between the ratios of individual residues regardless of the titration point (Supplementary Figure S1B). Further, subsets of residues with intensity loss more than one standard deviation below the mean were not uniform between the titration points, nor did they form a contiguous interaction surface on the structure (Supplementary Figure S1B). We thus infer that the observed intensity loss mostly originates from the molecular weight increase and global exchanges upon complex formation, which dominates possible line broadening at the contact surface. Taken together, our NMR titration experiment strongly supports a direct interaction between AcrIF7 and Cas8f–Cas5f, though it did not unambiguously identify the binding interface of AcrIF7.

**Table 1.** ITC analyses of binding between AcrIF7 and Cas8f–Cas5f mutants^a^

AcrIF7	Cas8f–Cas5f	*K*_D_ (nM)	*N*	Δ*G* (kcal/mol)	Δ*H* (kcal/mol)	*T*Δ*S* (kcal/mol)
WT	WT	46 ± 14	0.78 ± 0.00	–10.0 ± 0.2	–17.1 ± 0.2	–7.1 ± 0.3
D13K	WT	2451 ± 528	0.72 ± 0.02	–7.7 ± 0.1	–14.2 ± 0.6	–6.5 ± 0.7
E18K	WT	49 ± 4	0.88 ± 0.00	–10.0 ± 0.1	–19.6 ± 0.1	–9.6 ± 0.1
E22K	WT	50 ± 19	0.75 ± 0.01	–10.0 ± 0.2	–15.6 ± 0.3	–5.6 ± 0.4
D28K/D29K	WT	179 ± 25	0.75 ± 0.00	–9.2 ± 0.1	–12.9 ± 0.1	–3.7 ± 0.2
E33K/E34K	WT	4854 ± 1053	0.90 ± 0.04	–7.3 ± 0.1	–9.5 ± 0.6	–2.3 ± 0.6
E46K/E47K	WT	46 ± 11	0.80 ± 0.00	–10.0 ± 0.2	–17.0 ± 0.2	–7.0 ± 0.2
D57K	WT	333 ± 116	0.77 ± 0.02	–8.9 ± 0.2	–12.7 ± 0.4	–3.8 ± 0.4
WT	K29E(Cas8f)	327 ± 43	0.83 ± 0.01	–8.8 ± 0.1	–14.5 ± 0.2	–5.7 ± 0.2
D13K	K29E(Cas8f)	N.B.^b^	N.B.	N.B.	N.B.	N.B.
E33K/E34K	K29E(Cas8f)	N.B.	N.B.	N.B.	N.B.	N.B.
WT	K248E(Cas8f)	1873 ± 132	0.78 ± 0.01	–7.8 ± 0.0	–16.0 ± 0.2	–8.2 ± 0.2
D13K	K248E(Cas8f)	N.B.	N.B.	N.B.	N.B.	N.B.
D28K/D29K	K248E(Cas8f)	N.B.	N.B.	N.B.	N.B.	N.B.
E33K/E34K	K248E(Cas8f)	N.B.	N.B.	N.B.	N.B.	N.B.
D57K	K248E(Cas8f)	N.B.	N.B.	N.B.	N.B.	N.B.
AcrIF2^c^	WT	7.2 ± 2.0	0.84 ± 0.00	–11.0 ± 0.2	–22.2 ± 0.1	–11.1 ± 0.2

^a^Raw ITC data are provided in Supplementary Figure S5.

^b^No binding: Integrated heats from the measurement were not sufficient to constrain the least squares fit derived from a one-site binding model for the titration.

^c^Ref. (24).

**Figure 2.**
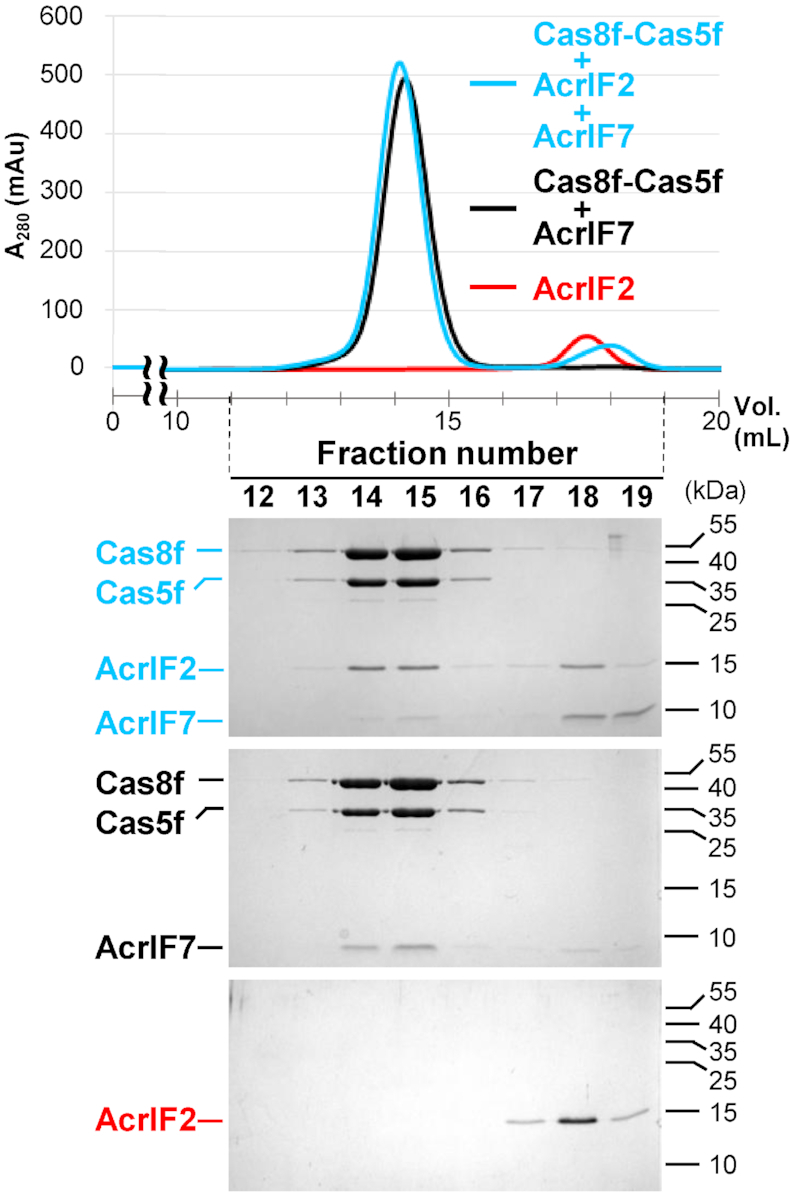
AcrIF7 competes with AcrIF2 for binding to the Cas8f–Cas5f heterodimer. Using analytical SEC, binding of AcrIF7 (20 μM) to the Cas8f–Cas5f heterodimer (20 μM) was tested with and without AcrIF2 (20 μM). In the presence of AcrIF2, most of AcrIF7 eluted separately from the Cas8f–Cas5f heterodimer, whereas the majority of AcrIF2 co-eluted with the heterodimer. Elution fractions were analyzed by SDS-PAGE. Uncropped gel images are shown in Supplementary Figure S9.

Since both AcrIF2 and AcrIF7 interact with the Cas8f–Cas5f heterodimer, we asked whether these Acr proteins also compete for the same binding interface on the Cas8f–Cas5f complex. In our analytical SEC experiment, we found that AcrIF7 did not associate tightly with the Cas8f–Cas5f heterodimer in the presence of AcrIF2 (Figure 2). While most of the AcrIF7 eluted separately from the Cas8f–Cas5f complex, the majority of the AcrIF2 co-eluted with the heterodimer. This observation is consistent with the fact that AcrIF2 (*K*_D_ = 7.2 nM) binds more strongly to the Cas8f–Cas5f heterodimer than AcrIF7 (*K*_D_ = 46 nM), indicating that these two Acr proteins have mutually exclusive binding interfaces on the Cas8f–Cas5f tail. In previous cryo-electron microscopy (EM) structures of the AcrIF2-bound Csy complex, AcrIF2 was found at the junction between Cas8f and Cas7f, but far from Cas5f (10,11). This suggests the binding interface for AcrIF7 is located on the Cas8f side of the Cas8f–Cas5f heterodimer.

### AcrIF7 adopts a novel α/β fold with a negatively charged surface

**Figure 3.**
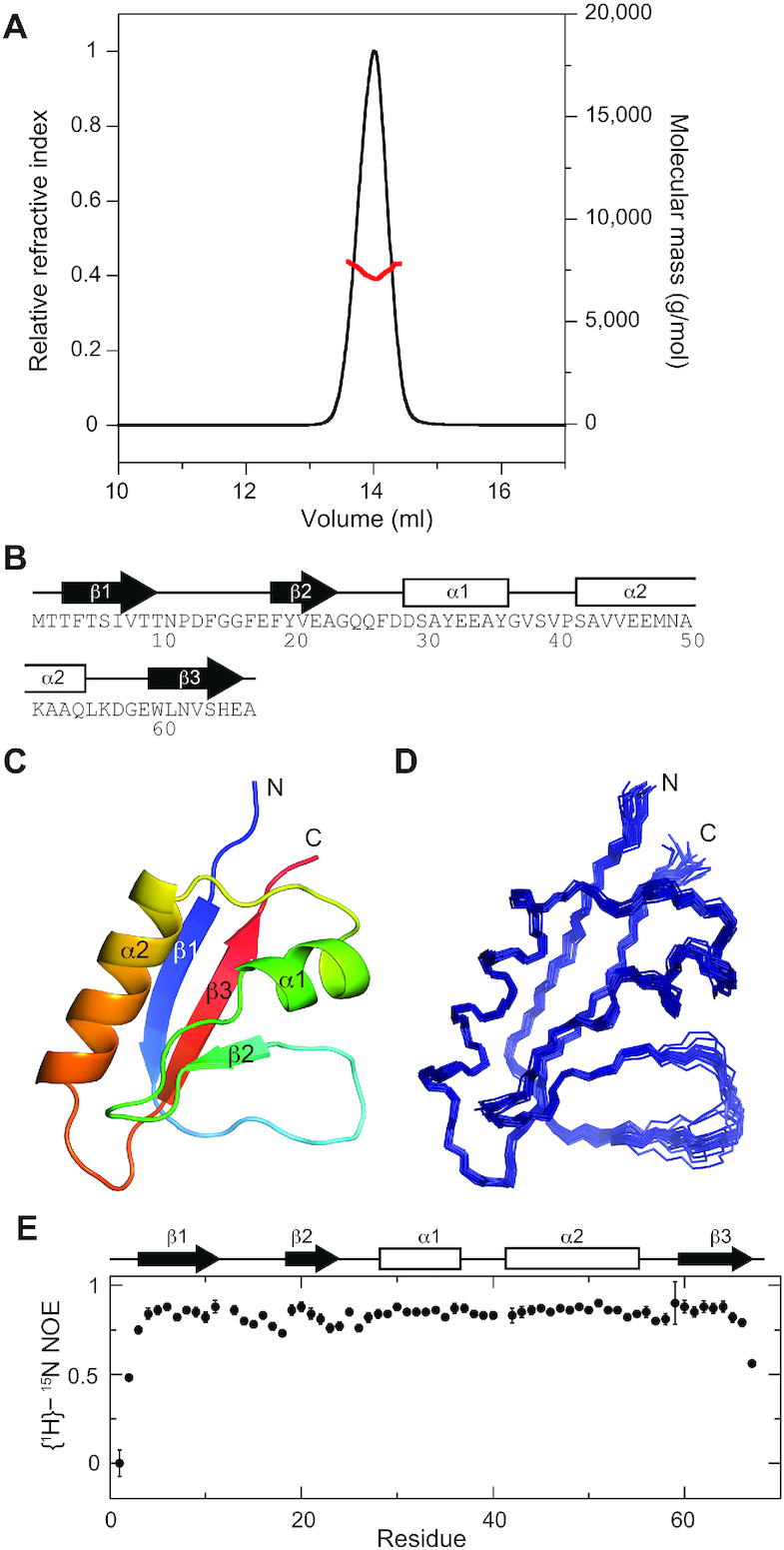
Structure and dynamics of AcrIF7. (**A**) SEC-MALS analysis of AcrIF7. The monomeric state of AcrIF7 in solution was detected by SEC-MALS. *Black* and *red* lines represent the normalized refractive index and the molecular mass of AcrIF7, respectively. The experimentally measured and theoretically calculated molecular masses of AcrIF7 are 7.5 and 7.3 kDa, respectively. (**B**) Schematic representation of secondary structural elements on the amino acid sequence of AcrIF7. (**C**) Solution structure of AcrIF7 as determined by NMR spectroscopy. The AcrIF7 structure is shown in *rainbow* format from the N terminus (*blue*) to the C terminus (*red*). (**D**) Superposition of the backbone atoms of the final 20 simulated annealing structures of AcrIF7. These structures are best-fit superposed on well-ordered secondary structures between residues 3–9, 19–22, 29–36, 42–54 and 60–66. (**E**) {^1^H}–^15^N heteronuclear NOE data as a function of residue number. The secondary structures of AcrIF7 are indicated above.

We found AcrIF7 (a.a. 1–67) emerges from SEC as a monomer, which is consistent with multi-angle light scattering (MALS) measurements (Figure 3A). The average molecular mass obtained from the light scattering and refractive index measurements was 7.5 ± 0.3 kDa. This is consistent with the calculated molecular mass of 7327.9 Da for the AcrIF7 monomer. The 2D ^1^H–^15^N heteronuclear single quantum correlation (HSQC) spectrum of AcrIF7 showed the well-dispersed backbone amide resonances typical of a compact folded protein (Supplementary Figure S2). Backbone and side chain ^1^H, ^15^N and ^13^C resonances were assigned using a suite of triple-resonance heteronuclear correlation NMR experiments. We obtained distance restraints from three-dimensional ^13^C-separated NOESY and ^15^N-separated NOESY experiments and then measured residual dipolar couplings (RDCs) in 11.5 mg/ml of *pf1* phage alignment medium. We solved the structure of AcrIF7 based on 1465 NMR restraints comprising 1244 experimental NOE restraints, 126 dihedral angle restraints, 64 backbone ^1^D_NH_ RDC restraints, and 31 hydrogen bonding restraints (Table 2).

**Table 2.** Restraints and structural statistics for AcrIF7

Experimental restraints	<SA>^a^
Nonredundant NOEs	1244
Dihedral angles, ϕ / ψ	63/63
Hydrogen bonds	31
Residual dipolar coupling, ^1^D_NH_	64
Total number of restraints	1465 (21.9 per residue)
Rms deviation from experimental restraints	
Distances (Å) (2032)	0.016 ± 0.004
Torsion angles (°) (196)	1.13 ± 0.10
Residual dipolar coupling *R*-factor (%)^b^	
^1^ *D*_NH_ (%) (64)	0.26 ± 0.01
Rms deviation from idealized covalent geometry	
Bonds (Å)	0.001 ± 0
Angles (°)	0.45 ± 0.01
Impropers (°)	0.37 ± 0.02
Coordinate precision (Å)^c^	
Backbone	0.60 ± 0.09
Heavy atoms	1.23 ± 0.09
Ramachandran statistics (%)	
Favored regions	94.9 ± 1.1
Allowed regions	5.1 ± 1.1

^a^For the ensemble of the final 20 simulated annealing structures.

^b^The magnitudes of the axial and rhombic components of the alignment tensor were 3.0 Hz and 0.34, respectively.

^c^Regions with secondary structures.

AcrIF7 features three β-strands that form an antiparallel β1–β3–β2 sheet with flanking α1 and α2 helices (Figure 3B). The β1 (residues 3–9) and β2 (residues 19–22) strands are connected by a long linker loop, followed by the α1 (residues 29–36) and α2 (residues 42–54) helices that each sit on the same side of the β-sheet opposite the β1–β2 loop (Figure 3C). The β3 strand (residues 60–66) is inserted between the β1and β2 strands in an antiparallel manner. We could not find structural homologs of AcrIF7 using the DALI program (DALI *Z*-score > 3.0), suggesting that AcrIF7 adopts a novel fold (25). When we deleted the long β1–β2 loop region of AcrIF7 and submitted the truncated coordinate as a search query, we essentially obtained the same results as the previous DALI run with the full-length structure. Overall secondary structures were well-defined in the 20 lowest-energy structures (Figure 3D). We note that the long β1–β2 loop (residues 10–18) exhibited a well-defined conformation, indicating an absence of dynamic motion. The {^1^H}–^15^N heteronuclear NOE values of the backbone amide groups further support that AcrIF7 adopts an overall rigid fold (Figure 3E). In particular, the NOE values of the β1–β2 loop are similar to those in the secondary structural regions, confirming the absence of loop dynamics (Figure 3E). We attribute this rigid loop conformation to the hydrophobic packing of Pro12 (β1–β2 loop) against Tyr20 (β2 strand) and Trp60 (β3 strand). We confirmed this hydrophobic packing when we observed unusual upfield shifts of Pro12 resonances (e.g. −0.785 ppm and −0.432 ppm for Hβ resonances) due to ring current effects from the aromatic side chains of Tyr20 and Trp60.

### Mutational analyses reveal key residues in Acr-Cas binding interface

**Figure 4.**
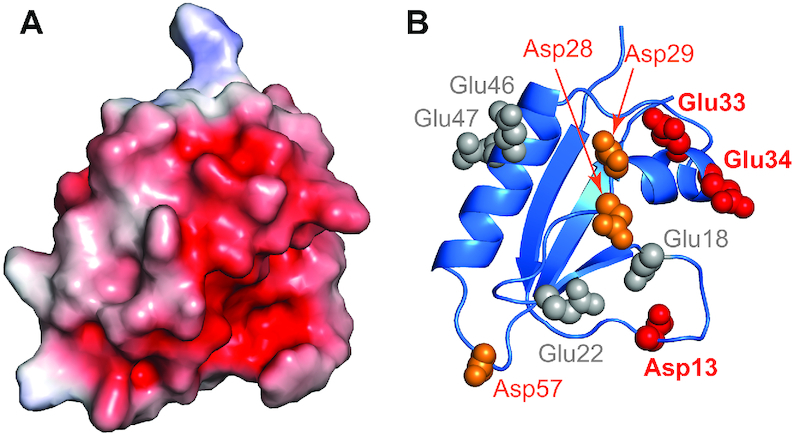
Negatively charged surface of AcrIF7. **(A**) Electrostatic potential surface of AcrIF7. PyMOL software (the PyMOL Molecular Graphics System, Version 2.0 Schrödinger, LLC.) was used with the Adaptive Poisson-Boltzmann Solver plugin to generate the surface (red = −5.0 kT, blue = +5.0 kT). (**B**) AcrIF7 structure as a cartoon diagram with carboxyl side chains shown as a space-filling model in the same orientation as the surface representation. Charge mutations of acidic residues caused varying impacts on the binding affinity of AcrIF7 to Cas8f–Cas5f, and the binding affinity contributions from each mutation site are color-coded as follows: large (*red*), small (*orange*) and negligible (*gray*).

The type I-F Acr proteins that target the Cas8f–Cas5f tail (AcrIF2, AcrIF6 and AcrIF10) reportedly associate with Cas8f via negatively charged interfaces (10–12). The electrostatic potential calculation of our AcrIF7 structure reveals a dense cluster of negatively charged Asp and Glu residues on its surface (Figure 4A), suggesting AcrIF7 also employs acidic surface residues to interact with Cas8f–Cas5f. To explore the role of electrostatic attraction in the interaction of AcrIF7 and Cas8f–Cas5f, we first tested whether their interaction is sensitive to salt concentration. In our ITC measurements, the strength of the interaction was reduced ∼2.7 fold when we increased the NaCl concentration from 150 to 500 mM (Supplementary Figure S3). This indicates that electrostatic attraction contributes to AcrIF7 binding to the Cas8f–Cas5f heterodimer.

To further investigate the electrostatic nature of this interaction, we generated seven AcrIF7 mutants in which one or two consecutive negatively-charged residues (Asp or Glu) were replaced with positively charged lysines (Figure 4B). We then tested the interactions of these mutants with the Cas8f–Cas5f heterodimer. We found that the mutant proteins exhibit CD spectra similar to WT AcrIF7 (Supplementary Figure S4). This indicates that the mutations did not disrupt the backbone fold. In our ITC analyses (Table 1 and Supplementary Figure S5), two AcrIF7 mutants (D13K and E33K/E34K) exhibited significant increases (50–100-fold) in *K*_D_ compared to the WT protein, suggesting that the mutated residues play crucial roles in the interaction with Cas8f–Cas5f. Two other mutants (D28K/D29K and D57K) showed relatively modest increases (4–7-fold) in *K*_D_, implying that these residues contribute more weakly to Cas8f–Cas5f binding. The three remaining mutants (E18K, E22K, and E46K/E47K) displayed binding affinities that were essentially identical to that of WT AcrIF7. Thus, it is unlikely that these four Glu residues participate in AcrIF7’s interaction with Cas8f–Cas5f. Our analytical SEC experiments with these AcrIF7 mutants also produced consistent results (Supplementary Figure S6).

We next introduced mutations into the Cas8f–Cas5f heterodimer. We focused on two Lys residues (Lys29 and Lys248) in Cas8f. These residues are conserved between the *X. albilineans* and *P. aeruginosa* homologs (Supplementary Figure S7), and the corresponding residues in *P. aeruginosa* Cas8f are reportedly involved in its interactions with other Cas8f-binding Acr proteins (namely, AcrIF2, AcriF6, and AcrIF10) (10–12). When Lys29 and Lys248 of Cas8f were mutated to negatively charged Glu residues, the binding affinity between AcrIF7 and the heterodimer dropped 7- and 40-fold, respectively (Table 1). This suggests the involvement of these positively charged Cas8f residues in AcrIF7 binding. The K29E and K248E mutants did not produce measurable isotherms in the ITC experiments with the AcrIF7 mutants (D13K, D28K/D29K, E33K/E34K and D57K) (Table 1). Our analytical SEC analyses also confirmed a lack of binding between these mutants (Supplementary Figure S6).

Together, our ITC and SEC analyses using mutant proteins demonstrate that electrostatic attraction is crucial for the binding of AcrIF7 with Cas8f–Cas5f. Notably, single mutations in each binding partner (e.g. D13K in AcrIF7 and K248E in Cas8f) can completely abolish the strong interaction when introduced together (Table 1), highlighting the critical roles of negatively charged carboxyl side chains in AcrIF7 and positively charged Lys residues in Cas8f.

### Molecular docking between AcrIF7 and Cas8f

**Figure 5.**
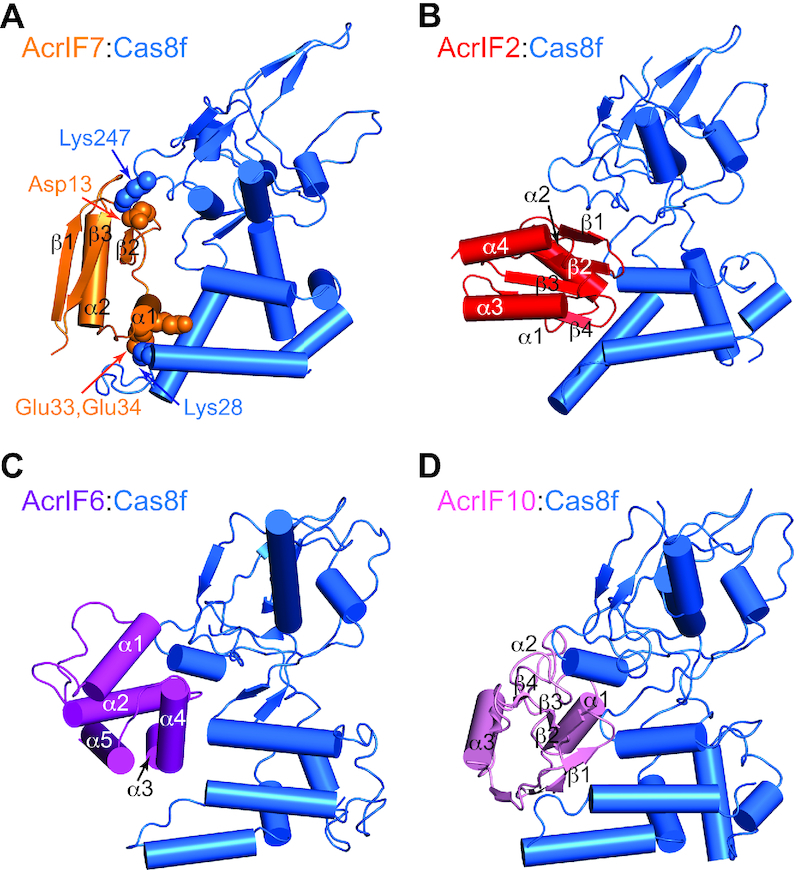
Structure comparison of type I-F Acr proteins in complex with *P. aeruginosa* Cas8f. Complex structure of Cas8f in *blue* with (**A**) AcrIF7 in *orange* (this study), (**B**) AcrIF2 in *red* (PDB code 6B47), (**C**) AcrIF6 in *purple* (PDB code 6VQX), and (**D**) AcrIF10 in *pink* (PDB code 6B48), in a cartoon representation. The structure of the AcrIF7:Cas8f complex was modeled based on the Csy complex structure and mutagenic studies, otherwise complex structures were determined by cryo-EM. The key residues for the interaction are annotated in the AcrIF7:Cas8f structure. All structures are shown in the same perspective. Only the N-terminal region of Cas8f (residues 1–267) responsible for the interaction is shown for visual clarity. Lys28 and Lys247 of *P. aeruginosa* Cas8f are equivalent to Lys29 and Lys248 of *X. albilineans* Cas8f, respectively.

We first report that our initial attempts to crystallize the AcrIF7-bound *X. albilineans* Cas8f-Cas5 heterodimer were unsuccessful. In particular, the *X. albilineans* Cas8f–Cas5 heterodimer was refractory to crystallization, which may be partly attributed to the conformational heterogeneity in the N-terminal hook domain of Cas8f as previously observed in the *P. aeruginosa* Csy complex structure (11). We enlisted the crystal screen conditions employed in our trial for the record in Supplementary Table S3. We then generated a docking model for the AcrIF7:Cas8f complex based on our AcrIF7 structure and the Cas8f subunit coordinate from the cryo-EM structure of the *P. aeruginosa* Csy complex (10). Residues with the largest impact on the binding affinity in our mutational analyses were used as distance restraints for the docking. In the final highest-score structure, AcrIF7 binds near the N-terminal hook region of Cas8f, which is also where other Cas8f-binding type I-F Acr proteins (AcrIF2, AcrIF6, and AcrIF10) interact (Figure 5) (10–12). Our model structure suggests that the binding site of AcrIF7 likely overlaps with that of AcrIF2, AcrIF6, and AcrIF10. This is consistent with the result of our competition SEC experiments using AcrIF2, AcrIF7, and Cas8f–Cas5f (Figure 2). The model structure also indicates a network of intermolecular hydrogen bonds and salt bridges, supporting the importance of electrostatic attractions in the association of AcrIF7 and Cas8f. In particular, the negatively charged surface of AcrIF7 interacts with highly-conserved positive charges of Cas8f required for target dsDNA recognition. Our data collectively predicts that AcrIF7 functions as a DNA mimic for the inhibition of type I-F CRISPR–Cas activity.

## DISCUSSION

In this study, we report the solution structure for AcrIF7 and characterize its interaction with Cas8f of the Csy complex. Acr proteins that inhibit the type I CRISPR–Cas system have been identified against subtypes I-B, I-C, I-D, I-E and I-F. Of these, the type I-F Acrs are the best characterized, comprising 15 distinct sequences. The type I-F Acrs generally associate with the Csy complex to hinder its recognition of target dsDNA or the formation of a crRNA–DNA heteroduplex. AcrIF3 is an exception, as it binds to Cas2/3 nuclease and disables its access to the Csy complex. To date, eight type I-F Acrs are known to directly bind to the Csy complex (Supplementary Table S1). AcrIF1, AcrIF8 and AcrIF9 associate with the Cas7f backbone (10,12), whereas AcrIF2, AcrIF6, AcrIF7 and AcrIF10 mainly associate with the Cas8f tail of the Csy complex (10–12). AcrIF4 binds to the Csy complex (26), but its target Cas component remains unknown. Acrs are unique in their sequences and structures, so that their targets and structural mechanisms are difficult to predict based on sequence alone. We note, however, that Acrs targeting Cas8f are highly negatively charged (p*I* < 4) to mimic the surface of target DNA, and that they bind to the Csy complex in a 1:1 stoichiometry. In contrast, Acrs that target the Cas7f backbone (and its associated crRNA) are more variable in their charges and stoichiometry: two copies of AcrIF1 (p*I* = 8.0) and AcrIF9 (p*I* = 7.8) sit along the Cas7f backbone in tandem, whereas a single AcrIF8 (p*I* = 5.4) binds to Cas7f on its own. In light of this observation, we anticipate that AcrIF13 (p*I* = 4.2) and AcrIE4-IF7 (p*I* = 4.3) may also target Cas8f to inhibit CRISPR–Cas nuclease activity. It is tempting to speculate that AcrIE4-IF7 elicits a dual Acr activity against type I-E and I-F CRISPR–Cas systems by mimicking the surface charges of DNA to target both Cas8e and Cas8f. We also predict that AcrIF5 (p*I* = 9.7) and AcrIF14 (p*I* = 8.4) bind to the Cas7f backbone in tandem, similar to AcrIF1.

We have identified key residues required for AcrIF7:Cas8f binding. The negative charges of Asp13, Glu33 and Glu34 on AcrIF7 were most important for Cas8f binding, and the positive charges of Lys29 and Lys248 on Cas8f were crucial for AcrIF7 binding. Expecting that these key residues participate in electrostatic interactions at the molecular interface, we modeled the structure of the AcrIF7:Cas8f complex based on the cryo-EM structure of the Csy complex (Figure 5A). Notably, AcrIF7 fits snugly into the surface of Cas8f in a way that is very similar to that of AcrIF2 (Figure 5A and B). Previous EM structures showed that AcrIF2, AcrIF6, and AcrIF10 targeted Cas8f at mutually exclusive binding interfaces (Figure 5B–D). Our study illustrates that all four I-F Acrs that target Cas8f bind to overlapping interfaces precluding the simultaneous binding of another Acr. AcrIF2 (α1α2β1β2β3β4α3α4), AcrIF7 (β1β2α1α2β3) and AcrIF10 (β1β2β3β4α1α2α3) contain antiparallel β-strands and flanking α-helices, whereas AcrIF6 (α1α2α3α4α5) contains only α-helices. A close examination of the electrostatic potential for the interaction surfaces reveals that these Acrs employ a cluster of negative charges to engage with the highly basic surface of Cas8f (Supplementary Figure S8). The key interfaces of AcrIF7 were located at the β1–β2 loop (Asp13) and the α1 helix (Glu33 and Glu34) (Figure 5A). AcrIF2 employed β1 and β2 strands along with the β1–β2 and β3–β4 connecting loops (Figure 5B), whereas AcrIF10 employed β3 and β4 strands as well as the β1–β2 loop as interfaces for Cas8f binding (Figure 5D). On the other hand, AcrIF6 interacted with Cas8f mainly via α1, α2 and α4 helices (Figure 5C). It is thus remarkable that these Acrs can target the same interface on Cas8f without sharing a common sequence or structural motif.

We note that the aforementioned I-F Acrs generally recognize Lys248 of *X. albilineans* Cas8f (referred to hereafter as Lys247 because of its position in the *P. aeruginosa* Cas8f sequence to avoid confusion with published Csy complex structures) as a common interface (Figure 5). The Csy complex structure suggests that Lys247 plays an important role in the interaction between Cas8f and the protospacer adjacent motif (PAM) region of dsDNA to discriminate self from non-self target sequences (11). In addition, using a multiple sequence alignment, we located Lys247 within the region that was most conserved among Cas8f homologs (Supplementary Figure S7). It was previously reported that a single charge mutation that alters the charge of Lys247 dramatically reduced the binding of the Csy complex to target dsDNA (10). This same mutation also significantly attenuated the binding of the Csy complex to AcrIF2 (10) and AcrIF7 (this study). Further, AcrIF6 mutations that disrupt the binding interface for Lys247 of Cas8f impaired the inhibition of the Csy complex in the target DNA cleavage assay (12). Taken together, we infer that the PAM interaction (PI) site of Cas8f is likely a common target of Acrs that imitate the negative charge distribution of dsDNA. These PI-targeting Acrs may have evolved to shape similar patterns of surface charges exploiting widely divergent sequences and fold landscapes. Given that Cas8 (or Cas10d for type I-D) is present across all type I CRISPR–Cas systems (27), PI-targeting may be a general strategy of Acrs that inhibit other than the I-F subtype. Indeed, a current inventory of published Acrs lists 11 different I-B, I-C, I-D and I-E Acrs that are largely acidic and potential PI blockers of the Cas8 subunit for each subtype.

PI-targeting is a well-documented mechanism of Acrs that inhibit the Cas9 nuclease of the class 2 CRISPR–Cas system. Both AcrIIA2 (p*I* = 4.1) and AcrIIA4 (pI = 4.2) directly bind to the PI domain of type II-A Cas9, preventing target dsDNA binding (28,29). These two type II-A Acrs lack sequence and structural similarity, suggesting a convergent inhibition mechanism similar to that of type I-F Acr proteins (AcrIF2 and AcrIF7) in this study. Previous analyses reported that *acrIIA2* and *acrIIA4* genes were mutually exclusive in the type II-A *acr* loci of *Listeria monocytogenes* prophages (30). In contrast, each of *acrIIA2* and *acrIIA4* genes frequently co-occurred with the *acrIIA1* gene coding for AcrIIA1 that inhibits the type II-A CRISPR–Cas system via a completely different mechanism (30–32). This might reflect the evolutionary pressure to remove a redundant defense system that could impose a fitness cost. We examined whether *acrIF2*, *acrIF6*, *acrIF7* and *acrIF10* genes were also exclusive to one another in the phage and prophage genomes. We employed the basic local alignment search tool (BLAST) to find the *acr* homologs in the complete prokaryotic genome, plasmids, and phage genome databases. The search found several hits of *acrIF2*, *acrIF6* and *acrIF7* in the prokaryotic and phage genome databases, but returned no hit in in the plasmid database (Supplementary Tables S4 and S5). Also, no search hit of *acrIF10* was detected in any of the three genome databases. We discovered that *acrIF2* and *acrIF7* were indeed mutually exclusive in the prokaryotic and phage genomes (Supplementary Tables S4 and S5). Unexpectedly, we did find a case of co-occurrence of *acrIF2* and *acrIF6* in the chromosome of *P. aeruginosa* strain CCUG 51971 (accession NZ_CP043328.1, Supplementary Table S4). Our observations generally support the viewpoint that phages do not deploy multiple Acr proteins with a similar mode of inhibition. Notwithstanding, the co-occurrence of functionally redundant *acrIF2* and *acrIF6* also suggests a possible scenario in which the seemingly redundant Acrs may work together in synergy for phage survival. It has been postulated that the intense arms race between bacteria and phages possibly lead to the development of bacterial defense mechanisms inactivating Acr proteins (12,33). The existence of redundant Acrs could be advantageous to phages in evading such bacterial anti-Acr systems. We also do not rule out possible moonlighting of Acr proteins for phage protection other than the CRIPSR inhibition. Functional investigations such as plaquing assays and lysogen analysis combined with different *acr* combinations may help to understand the biological relevance of the Acr redundancy.

In summary, we have determined a novel fold of AcrIF7 and identified its target as the Cas8f tail of the Csy complex. We have demonstrated that Acr proteins use diverse folds to target the PAM recognition site of Cas8f to block dsDNA binding, and this seems to be a general mechanism of action among type I-F Acrs. The PI-targeting Acrs would not function simultaneously when they compete for overlapping interface on a common target. Nonetheless, deploying a multitude of PI-targeting Acrs may contribute to the fitness for phage survival, potentially overcoming the escape mutations of host proteins.

## DATA AVAILABILITY

The atomic coordinates of the solution structure of AcrIF7 and the NMR restraints have been deposited in the Protein Data Bank (PDB code 6M3N) and the Biological Magnetic Resonance Bank (Accession code 36320), respectively.

## Supplementary Material

gkaa690_Supplemental_File

## Notes

Present address: Iktae Kim, Department of Biochemistry and Biophysics, Texas A&M University, College Station, TX 77843, USA.

Present address: Suji Hong and Donghyun Ka, LG Chem, Ltd., LG Science Park, Seoul 07796, Korea.
