# Supplementary material for: Structural and mechanistic insights into the CRISPR inhibition of AcrIF7
Source: Nucleic Acids Res. 2020 Aug 18;48(17):9959–68. doi: 10.1093/nar/gkaa690 (PMC7515697; doi:10.1093/nar/gkaa690)
Supplement: gkaa690_Supplemental_File [file gkaa690_supplemental_file.pdf]

Supplementary Information

Structural and mechanistic insights into the CRISPR inhibition of AcrIF7

Iktae Kim, Jasung Koo, So Young An et al.

Table S1. Summary of Acr proteins inhibiting type I-F CRISPR-Cas systems

Acr names	Number of amino acids	Theoretical pI[a]	Main target	Reference(s)
AcrIF1	78	8.03	Cas7f backbone	(1–5)
AcrIF2	96	3.98	Cas8f-Cas5f tail	(1–6)
AcrIF3	139	4.91	Cas2/3 nuclease	(1,2,7)
AcrIF4	100	5.25	Csy complex	(1,2)
AcrIF5	79	9.69	Unknown	(1)
AcrIF6	100	3.87	Cas8f-Cas5f tail	(8,9)
AcrIF7	67	3.88	Cas8f-Cas5f tail	(8), This study
AcrIF8	92	5.36	Cas7f backbone	(8,9)
AcrIF9	68	7.78	Cas7f backbone	(8–10)
AcrIF10	97	3.99	Cas8f-Cas5f tail	(5,8)
AcrIF11	132	4.38	Unknown	(11)
AcrIF12	124	6.07	Unknown	(11)
AcrIF13	115	4.17	Unknown	(11)
AcrIF14	124	8.37	Unknown	(11)
AcrIE4-IF7	119	4.27	Unknown	(11)

[a]Calculated by the ProtParam tool (https://web.expasy.org/protparam)

Table S2. Primers used in this study

Primer		5′-3′ Sequence	Description
(His)$_6$-MBP-AcrIF7	Forward	ATTTCCAGGGCCATATGACAACATTTACCTCAATAGTAACC	Cloning into pET28a vector
	Reverse	GGTGGTGGTGCTCGAGTTATGCCTCATGACTCACATTCAG	
AcrIF7 D13K	Forward	GTAACCACTAATCCGAAATTTGGCGGGTTCGAG TTT TAC GTC	Site-directed mutagenesis
	Reverse	GACGTAAAACTCGAACCCGCCAAATTTCGGATTAGTGGTTAC	
AcrIF7 E18K	Forward	CCGGATTTTGGCGGGTTCAAATTTTACGTCGAAGCAGGT	Site-directed mutagenesis
	Reverse	ACCTGCTTCGACGTAAAATTTGAACCCGCCAAAATCCGG	
AcrIF7 E22K	Forward	GGGTTCGAGTTTTACGTCAAAGCAGGTCAGCAATTTGATGATTCC	Site-directed mutagenesis
	Reverse	GGAATCATCAAATTGCTGACCTGCTTTGACGTAAAACTCGAACCC	
AcrIF7 D28K/D29K	Forward	GTCGAAGCAGGTCAGCAATTTAAAAAATCCGCGTACGAAGAGGCG	Site-directed mutagenesis
	Reverse	CGCCTCTTCGTACGCGGATTTTTTAAATTGCTGACCTGCTTCGAC	
AcrIF7 E33K/E34K	Forward	GGTCAGCAATTTGATGATTCCGCGTACAAAAAAGCGTATGGTGTG	Site-directed mutagenesis
	Reverse	CACACCATACGCTTTTTTGTACGCGGAATCATCAAATTGCTGACC	
AcrIF7 E46K/E47K	Forward	CCTTCGGCAGTCGTTAAAAAAATGAACGCTAAAGCAGCGCAG	Site-directed mutagenesis
	Reverse	CTGCGCTGCTTTAGCGTTCATTTTTTTAACGACTGCCGAAGG	
AcrIF7 D57K	Forward	GCAGCGCAGCTGAAAAAAGGAGAATGGCTGAATGTGAG	Site-directed mutagenesis
	Reverse	CTCACATTCAGCCATTCTCCTTTTTTCAGCTGCGCTGC	
Cas8f K29E	Forward	CGGCGTGAGGCCGAACTGAAGGGGAATGAG	Site-directed mutagenesis
	Reverse	CTCATTCCCCTTCAGTTCGGCCTCACGCCG	
Cas8f K248E	Forward	CTTGGTGGTACCGAACCGCAGAATATCAGCCAGCTCAATAGC	Site-directed mutagenesis
	Reverse	GCTATTGAGCTGGCTGATATTCTGCGGTTCGGTACCACCAAG	

Table S3. Crystallization screens tested in this study

Product name	Company	Product number	Number of conditions
Structure Screen 1	Molecular Dimensions	MD1-01	50
Structure Screen 2	Molecular Dimensions	MD1-02	50
PACT premier	Molecular Dimensions	MD1-29	96
ProPlex	Molecular Dimensions	MD1-38	96
MIDASplus	Molecular Dimensions	MD1-106	96
Wizard Classic 1	Rigaku	EB-W1-T	48
Wizard Classic 2	Rigaku	EB-W2-T	48
Wizard Classic 3	Rigaku	EB-W3-T	48
Wizard Classic 4	Rigaku	EB-W4-T	48
MCSG-1 Crystal Screen	Anatrace	MCSG-1T	96
MCSG-2 Crystal Screen	Anatrace	MCSG-2T	96
MCSG-3 Crystal Screen	Anatrace	MCSG-3T	96
MCSG-4 Crystal Screen	Anatrace	MCSG-4T	96

Table S4. The basic local alignment search tool (BLAST) results of AcrIF7 (accession number: ACD38920.1), AcrIF2 (accession number: NP_938237), and AcrIF6 (accession number: WP_043884810) using the tblastn search engine (search translated nucleotide databases using a protein query) against microbial genome database in the National Center for Biotechnology Information (NCBI) web page (https://blast.ncbi.nlm.nih.gov/Blast.cgi?PAGE_TYPE=BlastSearch&BLAST_SPEC=MicrobialGenomes). We report the results of searches for each Acr protein against a *complete prokaryotic genomes* database in NCBI. The BLAST results were further examined manually and similar sequences were selected as hits only when the query coverage was greater than 50%, and the E-value was less than 0.01. The search of AcrIF10 (accession number: KEK29119) did not return any hit sequence. The *Pseudomonas aeruginosa* strain that contains both *acrIF2* and *acrIF6* genes is highlighted in yellow.

Acr Type	Description	Maximum alignment score	Query coverage	E-value	Percent identity	Accession
AcrIF7	Pseudomonas aeruginosa strain BAMCPA07-48, complete genome	171	100%	9.00E-48	98.8	NZ_CP015377.1
	Pseudomonas aeruginosa strain AR445 chromosome, complete genome	165	97%	7.00E-46	97.5	NZ_CP029088.1
	Pseudomonas aeruginosa strain RIVM-EMC2982 chromosome, complete...	165	97%	7.00E-46	97.5	NZ_CP016955.1
	Pseudomonas aeruginosa strain Carb01 63, complete genome	165	97%	7.00E-46	97.5	NZ_CP011317.1
	Pseudomonas aeruginosa SCV20265, complete sequence	160	98%	3.00E-44	93.9	NC_023149.1
	Pseudomonas aeruginosa strain NCTC11445 chromosome 1	108	96%	9.00E-26	62.5	NZ_LR134308.1
	Pseudomonas citronellolis strain SJTE-3 chromosome, complete...	73.6	77%	1.00E-13	57.8	NZ_CP015878.1
	Pseudomonas aeruginosa strain PB369 chromosome, complete genome	67.8	72%	2.00E-11	60.0	NZ_CP025049.1
	Pseudomonas aeruginosa strain PB368 chromosome, complete genome	67.8	72%	2.00E-11	60.0	NZ_CP025050.1
	Janthinobacterium svalbardensis strain PAMC 27463 chromosome,...	50.4	75%	2.00E-05	44.1	NZ_CP023422.1
	Janthinobacterium sp. SNU WT3 strain SNU WT1 chromosome,...	50.4	75%	2.00E-05	44.1	NZ_CP041185.1
	Janthinobacterium sp. LM6 chromosome, complete genome	50.4	75%	2.00E-05	44.1	NZ_CP019510.1
	Janthinobacterium agaricidamnosum strain BHSEK chromosome,...	50.1	86%	2.00E-05	44.3	NZ_CP033019.1
	Janthinobacterium lividum strain EIF1 chromosome, complete genome	49.3	69%	5.00E-05	46.0	NZ_CP048832.1

	Janthinobacterium lividum strain EIF2 chromosome, complete genome	49.3	69%	5.00E-05	46.0	NZ_CP049828.1
	Janthinobacterium sp. 1_2014MBL_MicDiv, complete genome	48.1	69%	1.00E-04	46.0	NZ_CP011319.1
AcrIF2	Pseudomonas aeruginosa strain DVT401 chromosome, complete genome	112	77%	4.00E-27	81.4	NZ_CP050335.1
	Pseudomonas aeruginosa strain IMP68 chromosome, complete genome	112	77%	6.00E-27	82.9	NZ_CP028849.1
	Pseudomonas aeruginosa strain IMP66 chromosome, complete genome	112	77%	6.00E-27	82.9	NZ_CP028959.1
	Pseudomonas aeruginosa strain IMP67 chromosome, complete genome	112	77%	6.00E-27	82.9	NZ_CP028848.1
	Pseudomonas aeruginosa strain CCUG 51971 chromosome, complete...	112	77%	6.00E-27	82.9	NZ_CP043328.1
	Pseudomonas aeruginosa strain Y82 chromosome, complete genome	112	77%	6.00E-27	82.9	NZ_CP030912.1
AcrIF6	Pseudomonas aeruginosa strain JB2 chromosome, complete genome	171	100%	1.00E-47	86.0	NZ_CP028917.1
	Pseudomonas aeruginosa strain AR_0353 chromosome, complete genome	171	100%	2.00E-47	86.0	NZ_CP027172.1
	Pseudomonas aeruginosa strain Pa58 chromosome, complete genome	171	100%	2.00E-47	86.0	NZ_CP021775.1
	Pseudomonas aeruginosa strain F63912, complete genome	170	100%	3.00E-47	85.0	NZ_CP008858.2
	Pseudomonas aeruginosa strain CCUG 51971 chromosome, complete...	170	100%	3.00E-47	85.0	NZ_CP043328.1

Table S5. The basic local alignment search tool (BLAST) results of AcrIF7 (accession number: ACD38920.1) and AcrIF2 (accession number: NP_938237) using the tblastn search engine (search translated nucleotide databases using a protein query) against microbial genome database in the National Center for Biotechnology Information (NCBI) web page (https://blast.ncbi.nlm.nih.gov/Blast.cgi?PAGE_TYPE=BlastSearch&BLAST_SPEC=MicrobialGenomes). We report the results of searches for each Acr protein against a *complete bacteriophage* database in NCBI. The BLAST results were further examined manually and similar sequences were selected as hits only when the query coverage was greater than 50%, and the E-value was less than 0.01. The search of AcrIF6 (accession number: WP_043884810) and AcrIF10 (accession number: KEK29119) did not return any hit sequence.

Acr Type	Description	Maximum alignment score	Query coverage	E-value	Percent identity	Accession
AcrIF7	Pseudomonas phage LPB1, complete genome	165	97%	3.00E-48	97.5	NC_027298.1
	Pseudomonas phage H70, complete genome	162	97%	4.00E-47	96.3	NC_027384.1
	Pseudomonas phage vB_PaeP_Tr60_Ab31	160	98%	1.00E-46	93.9	NC_023575.1
	Pseudomonas phage PA1/KOR/2010, complete genome	37.7	56%	0.003	38.3	NC_023700.1
	Pseudomonas phage MP48, complete genome	37.7	56%	0.003	38.3	NC_024782.1
AcrIF2	Pseudomonas phage JD024, complete genome	112	77%	3.00E-29	82.9	NC_024330.1
	Bacteriophage D3112, complete genome	112	77%	3.00E-29	82.9	NC_005178.1
	Pseudomonas phage MP29, complete genome	112	77%	3.00E-29	82.9	NC_011613.1

Figure S1. NMR titration experiments between AcrIF7 and Cas8f-Cas5f in 20 mM Tris-HCl, pH 7.5, 150 mM NaCl, and 1 mM TCEP. (**A**) Superimposed ^{1}H–^{15}N HSQC NMR spectra of free AcrIF7 (*black*) and AcrIF7 in complex with Cas8f-Cas5f heterodimer *(red*). (**B**) Changes in intensity ratios of backbone amide resonances of ^{15}N-AcrIF7 upon stoichiometric titrations with Cas8f-Cas5f according to the color code on the right. Error bars represent the uncertainty of peak integration, estimated from the r.m.s. noise in the signal-free region. Pale shades define the boundary of one standard deviation of the mean value for individual titrations in the same color code.

A

B

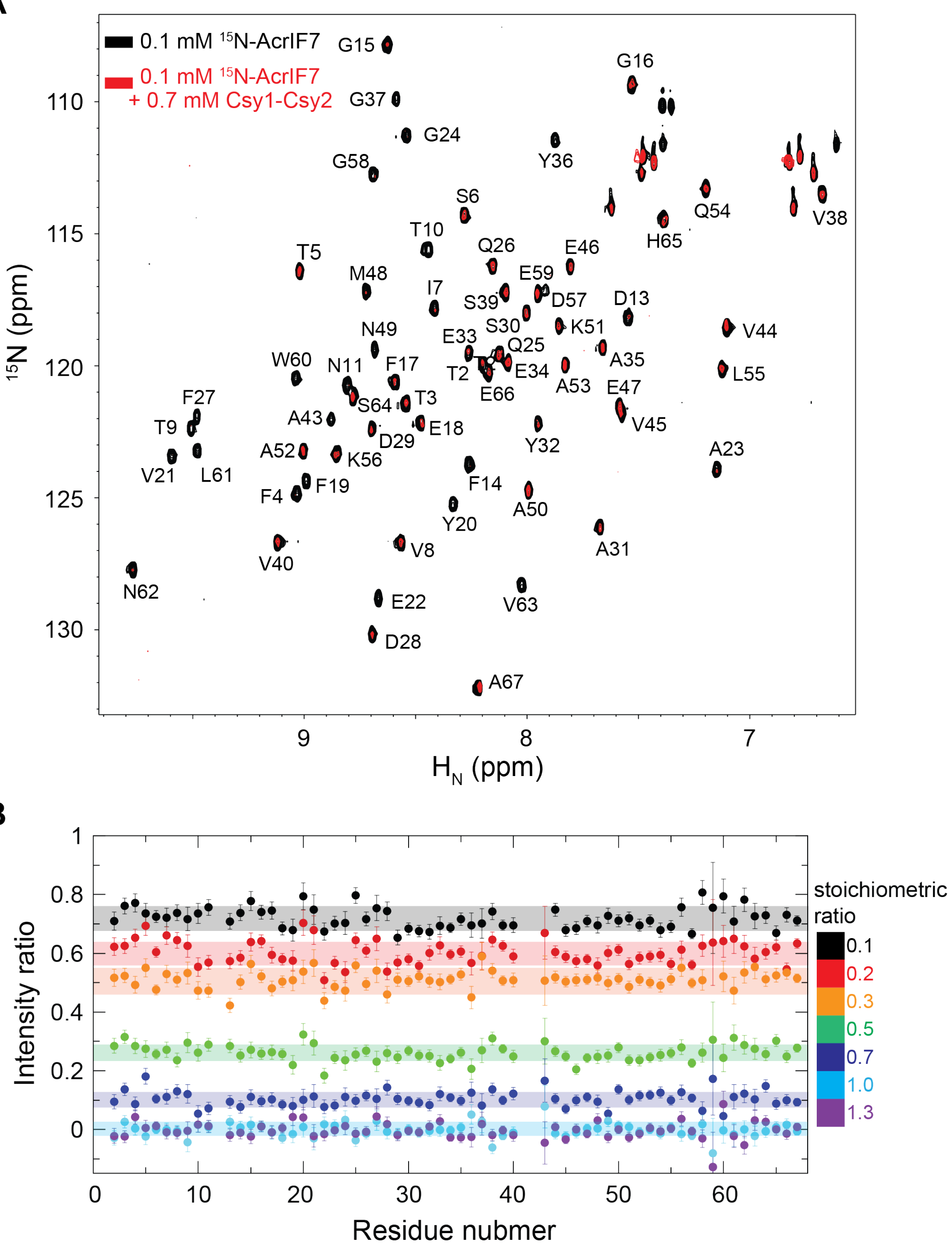

Figure S2. ^{1}H–^{15}N HSQC NMR spectrum of AcrIF7. The backbone amide resonances are annotated with the residue numbers. The HSQC spectrum is obtained in 20 mM HEPES, pH 7.0, 150 mM NaCl, and 2 mM DTT, which is the same buffer used for structure calculation. Slightly lower pH 7.0 in this condition compared to pH 7.5 used for the titration in Figure S1 reduced the solvent exchange rates of amide groups, and additional resonances of Met1 and Ser42 appeared in this spectrum. We note that Met1 is a penultimate residue in our construct, since removal of the His_6-MBP-tag by a TEV protease left a Gly residue before Met1 as an artefact. Accordingly, the amide resonance of Met1 is observed in the spectrum.

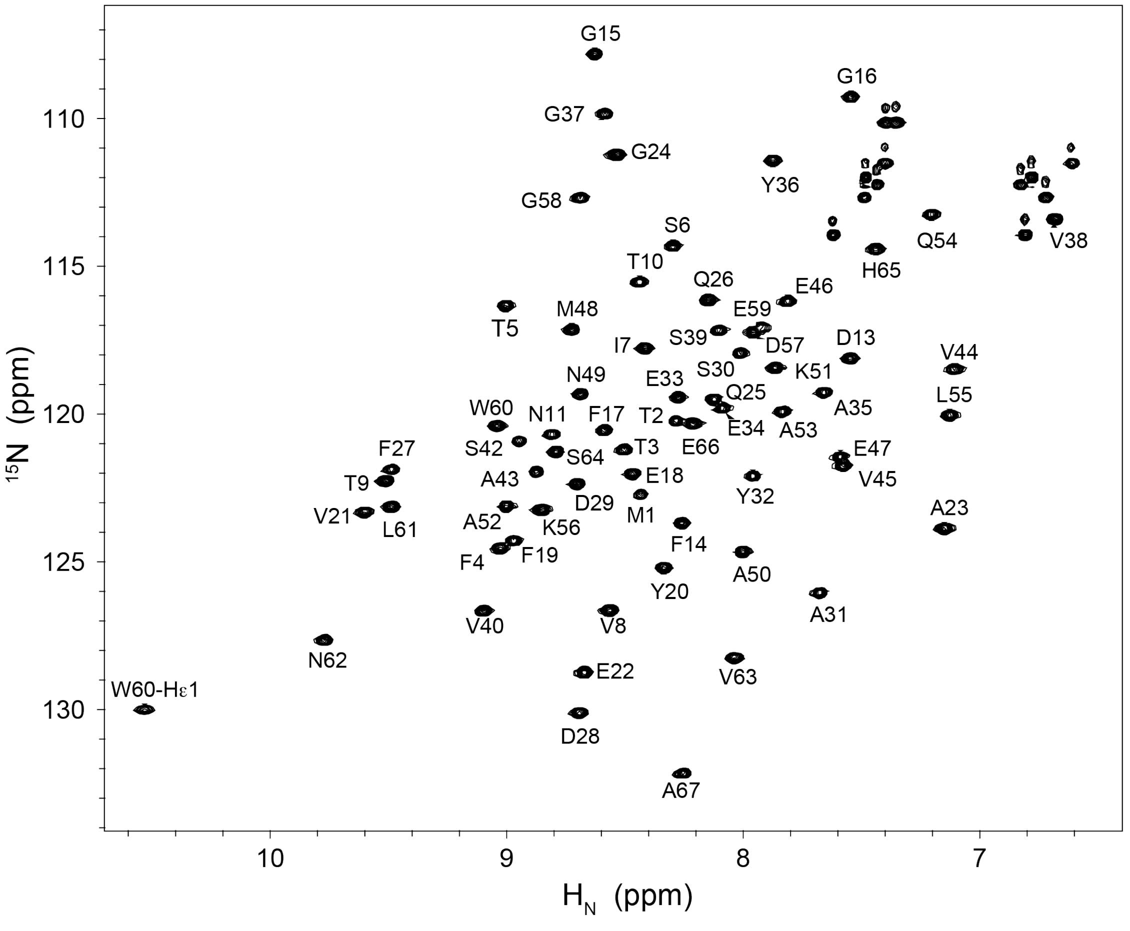

Figure S3. ITC analysis for the binding of AcrIF7 to Cas8f-Cas5f at 500 mM NaCl. The K_D and thermodynamic parameters are shown below.

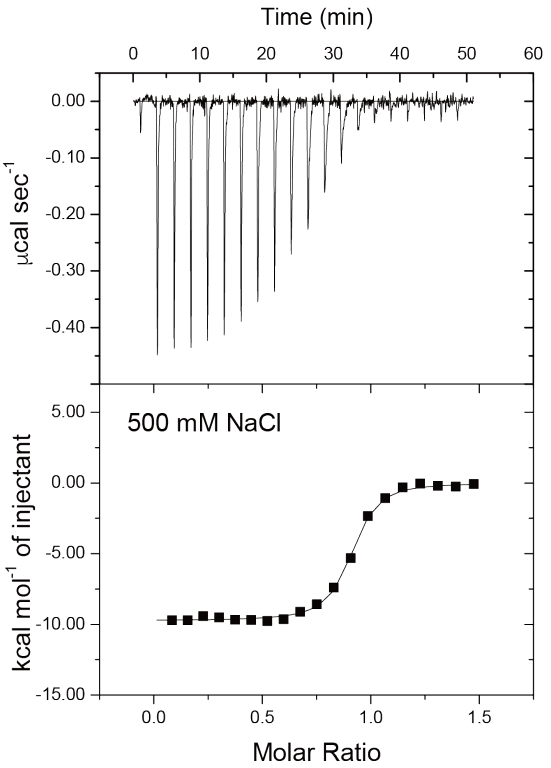

N	K_D (nM)	ΔG (kcal/mol)	ΔH (kcal/mol)	TΔS (kcal/mol)
0.9 ± 0.0	124 ± 13	–9.4 ± 0.1	–9.9 ± 0.1	–0.3 ± 0.1

Figure S4. CD spectra of AcrIF7 and Cas8f-Cas5f mutants. All mutants exhibited CD spectra similar to those of WT proteins, indicating that the charge reversal mutations did not perturb the secondary structures of individual mutants.

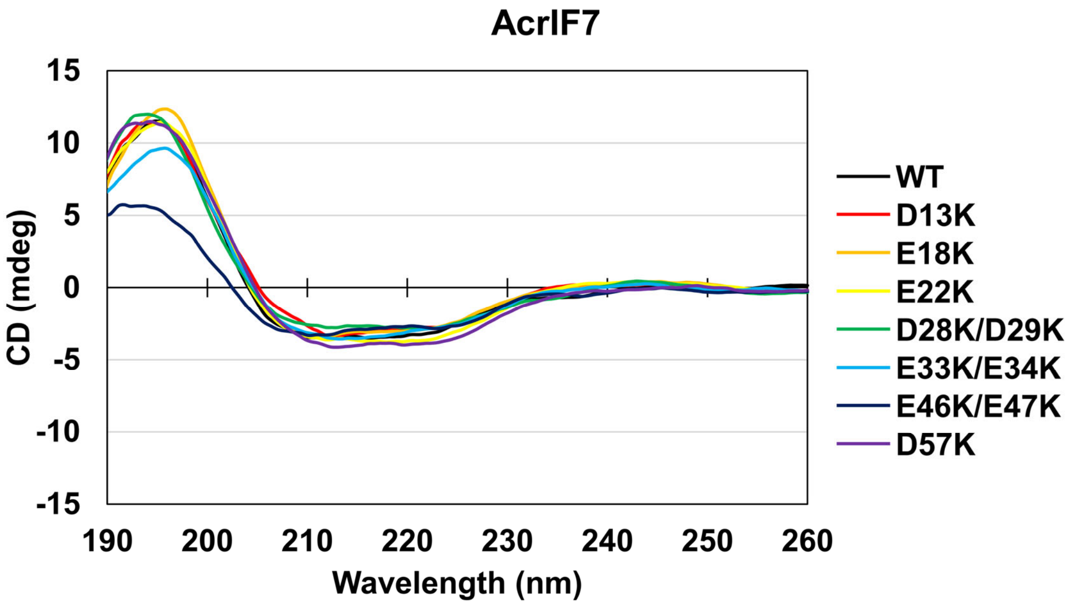

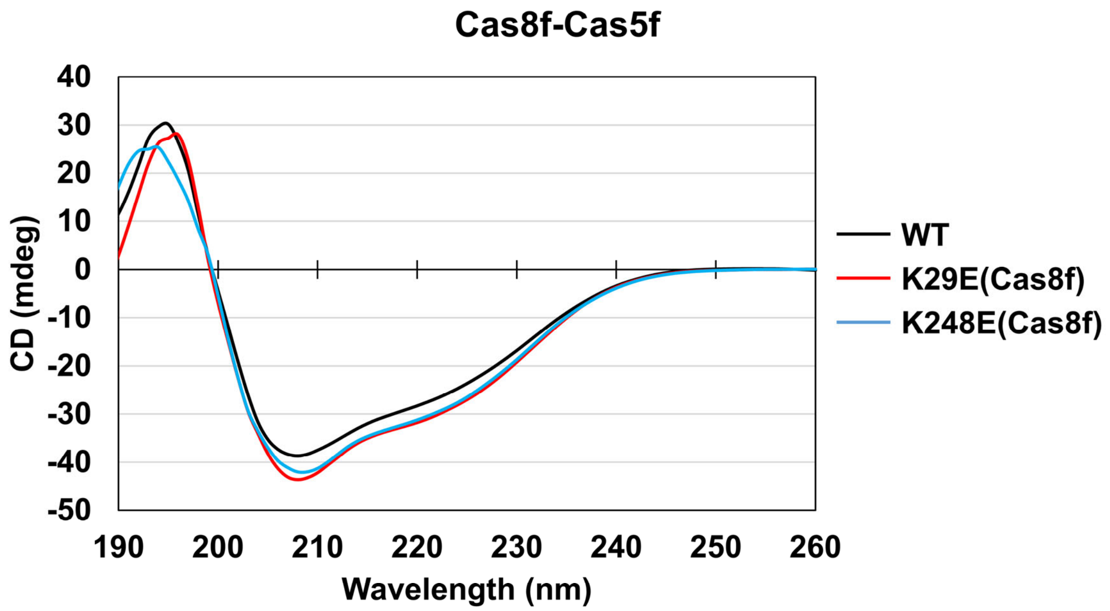

Figure S5. Integrated heats of injection for the interaction between AcrIF7 and Cas8f-Cas5f mutants calculated from ITC measurements. Titrations were carried out between WT Cas8f-Cas5f and (**A**) AcrIF7^{D13K}, (**B**) AcrIF7^{E18K}, (**C**) AcrIF7^{E22K}, (**D**) AcrIF7$^{D28K/D29K}$, (**E**) AcrIF7$^{E32K/E33K}$, (**F**) AcrIF7$^{E46K/E47K}$, and (**G**) AcrIF7^{D58K}. Titrations were also performed between WT AcrIF7 and (**H**) Cas8f^{K29E}-Cas5f, and (**I**) Cas8f^{K248E}-Cas5f. The equilibrium dissociation constants and thermodynamic parameters derived from the data are listed in Table 2 of the main text. The equilibrium dissociation constant (K_D) and the mutation site are shown above each isotherm.

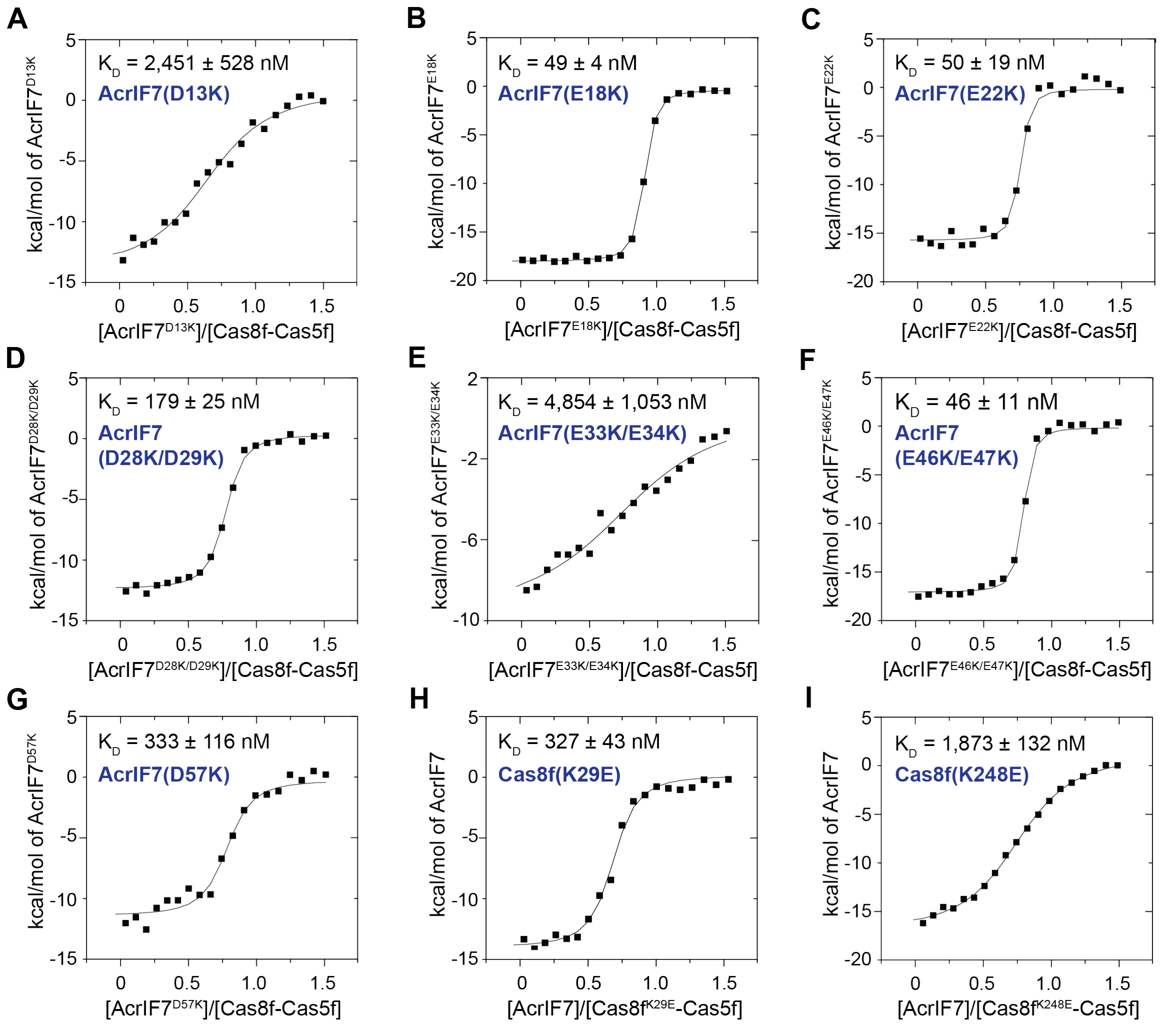

Figure S6. Analytical SEC analyses for testing interactions between AcrIF7 and Cas8f-Cas5f mutants**.** Elution fractions were analyzed by SDS-PAGE.

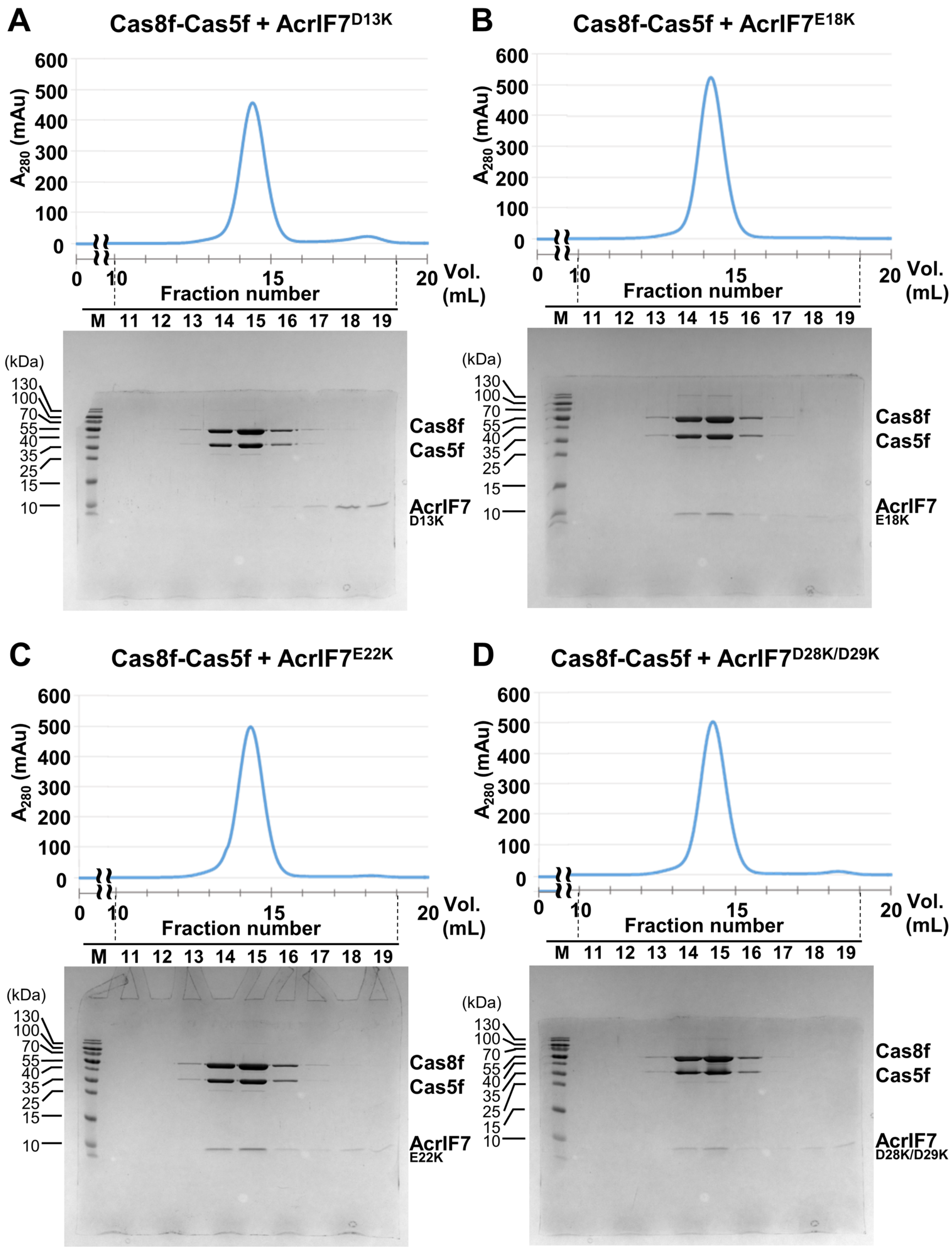

Figure S6. Analytical SEC analyses for testing interactions between AcrIF7 and Cas8f-Cas5f mutants**.** Elution fractions were analyzed by SDS-PAGE. (Continued)

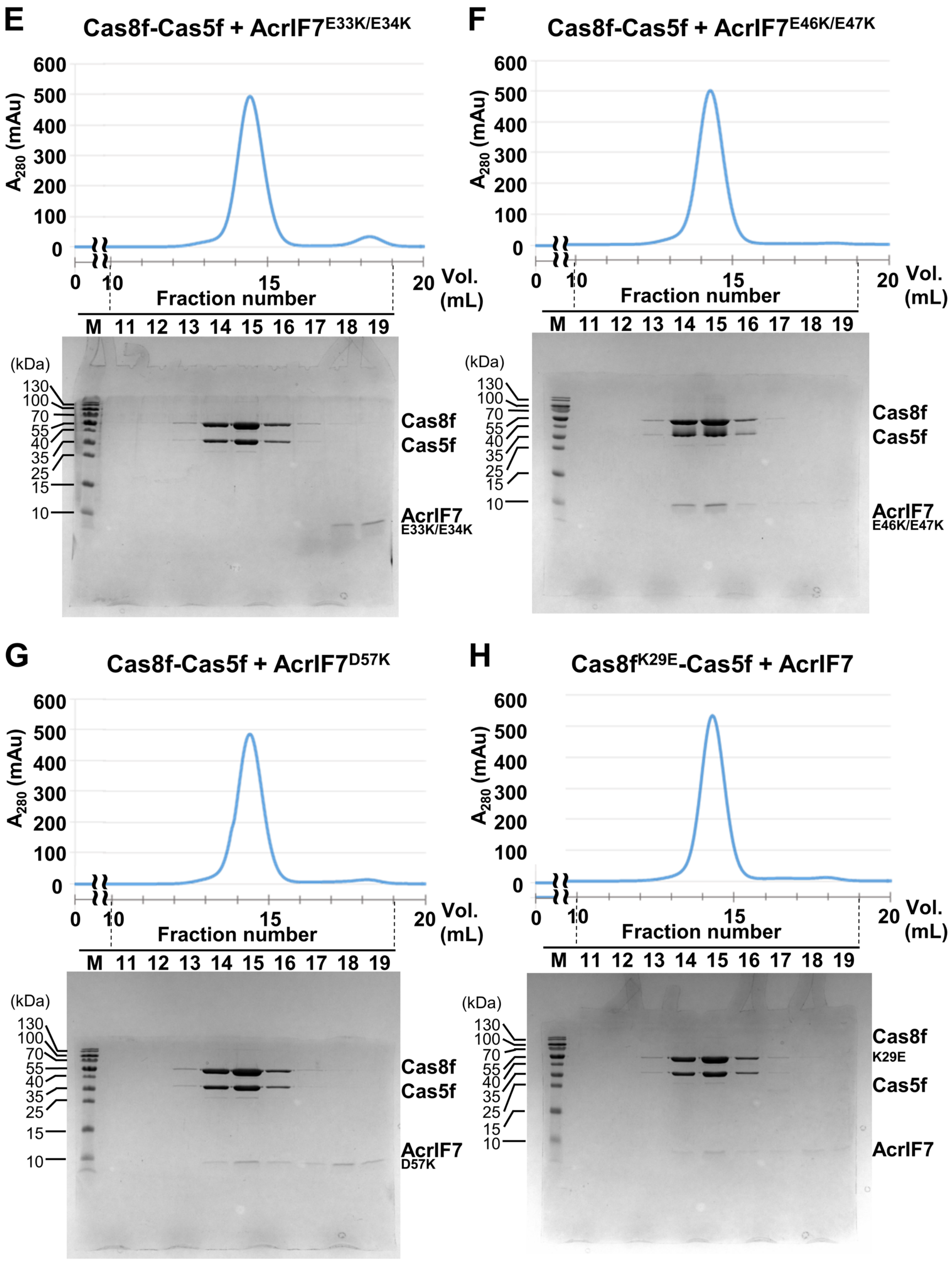

Figure S6. Analytical SEC analyses for testing interactions between AcrIF7 and Cas8f-Cas5f mutants**.** Elution fractions were analyzed by SDS-PAGE. (Continued)

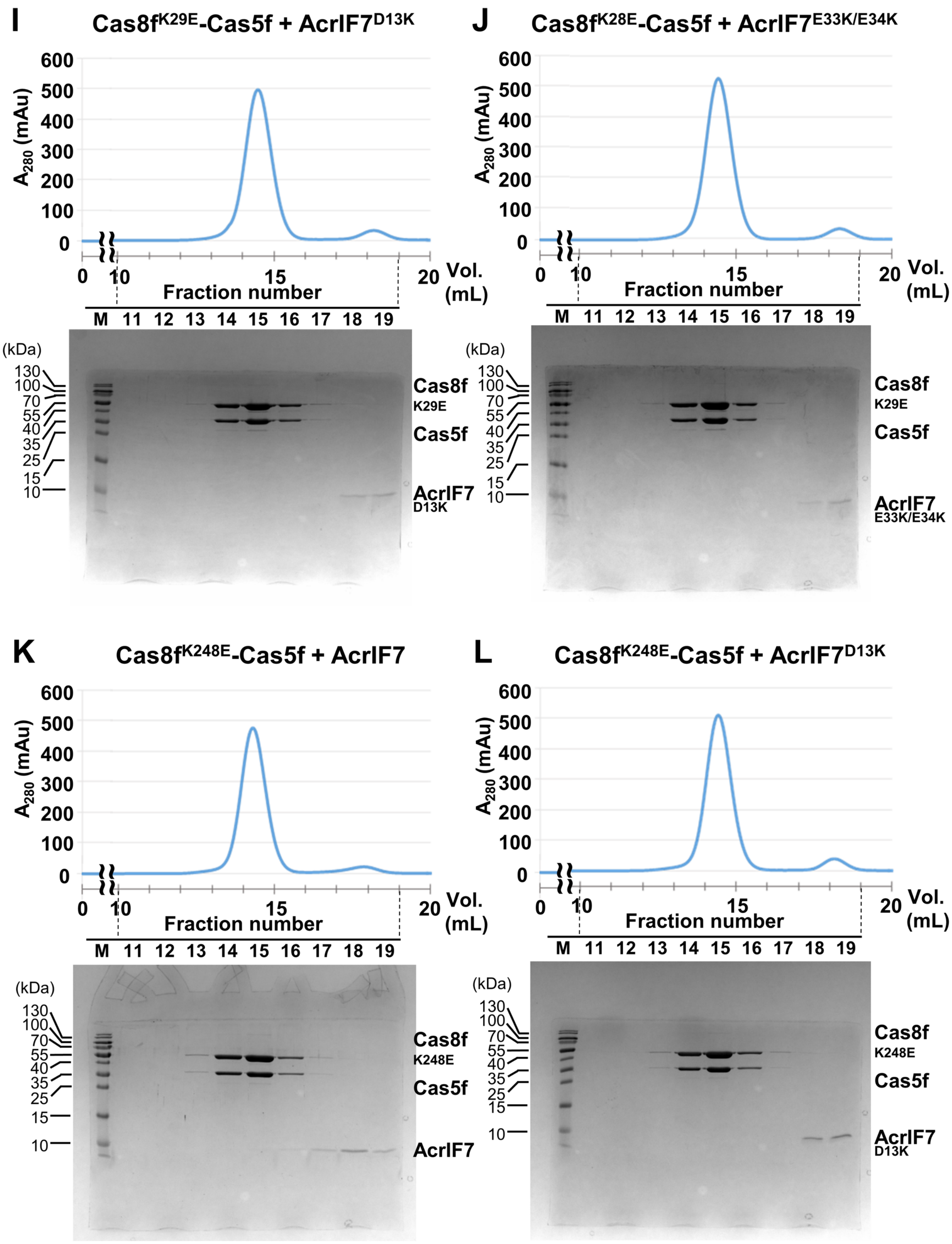

Figure S6. Analytical SEC analyses for testing interactions between AcrIF7 and Cas8f-Cas5f mutants**.** Elution fractions were analyzed by SDS-PAGE. (Continued)

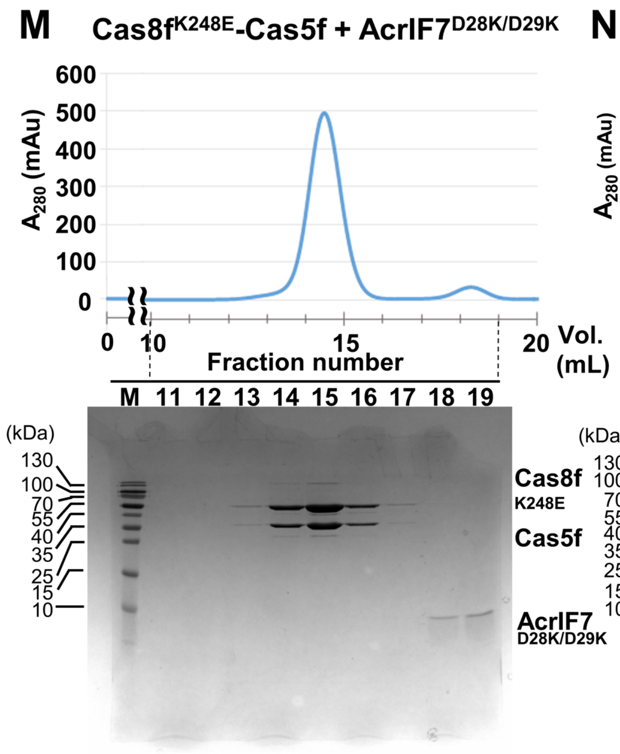

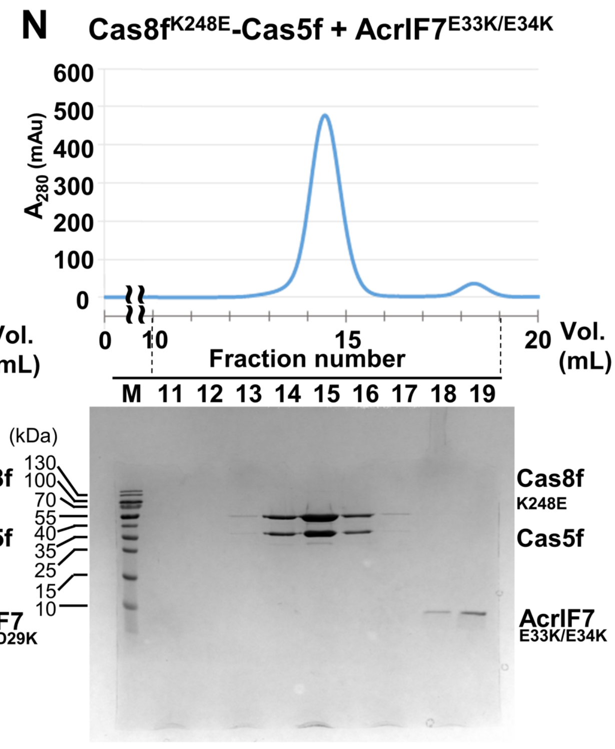

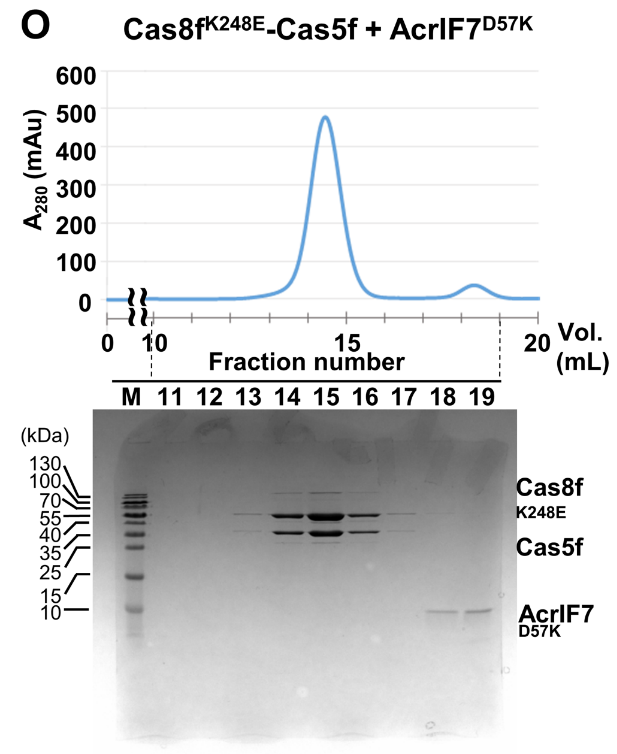

Figure S7. Multiple sequence alignment of Cas8f homologs. Lys29 and Lys248 of *X. albilineans* Cas8f are indicated as filled squares in *orange* above the sequence alignment. Full names of the host organisms and their accession IDs are as follows: *Pseudomonas aeruginosa* (WP_171978563.1), *Xanthomonas albilineans* (WP_045766478.1), *Methylococcus sp.* IM1 (WP_169602678.1), *Halomonas halmophila* (WP_170214888.1), *Leeia sp.* IMCC25680 (WP_168878644.1), *Marinomonas sp.* M1K-6 (WP_168826846.1), *Sporomusaceae bacterium* FL31 (WP_170978084.1), *Marinomonas sp.* UCMA 3892 (WP_169460117.1), and *Sphaerochaeta coccoides* (WP_013739764.1).

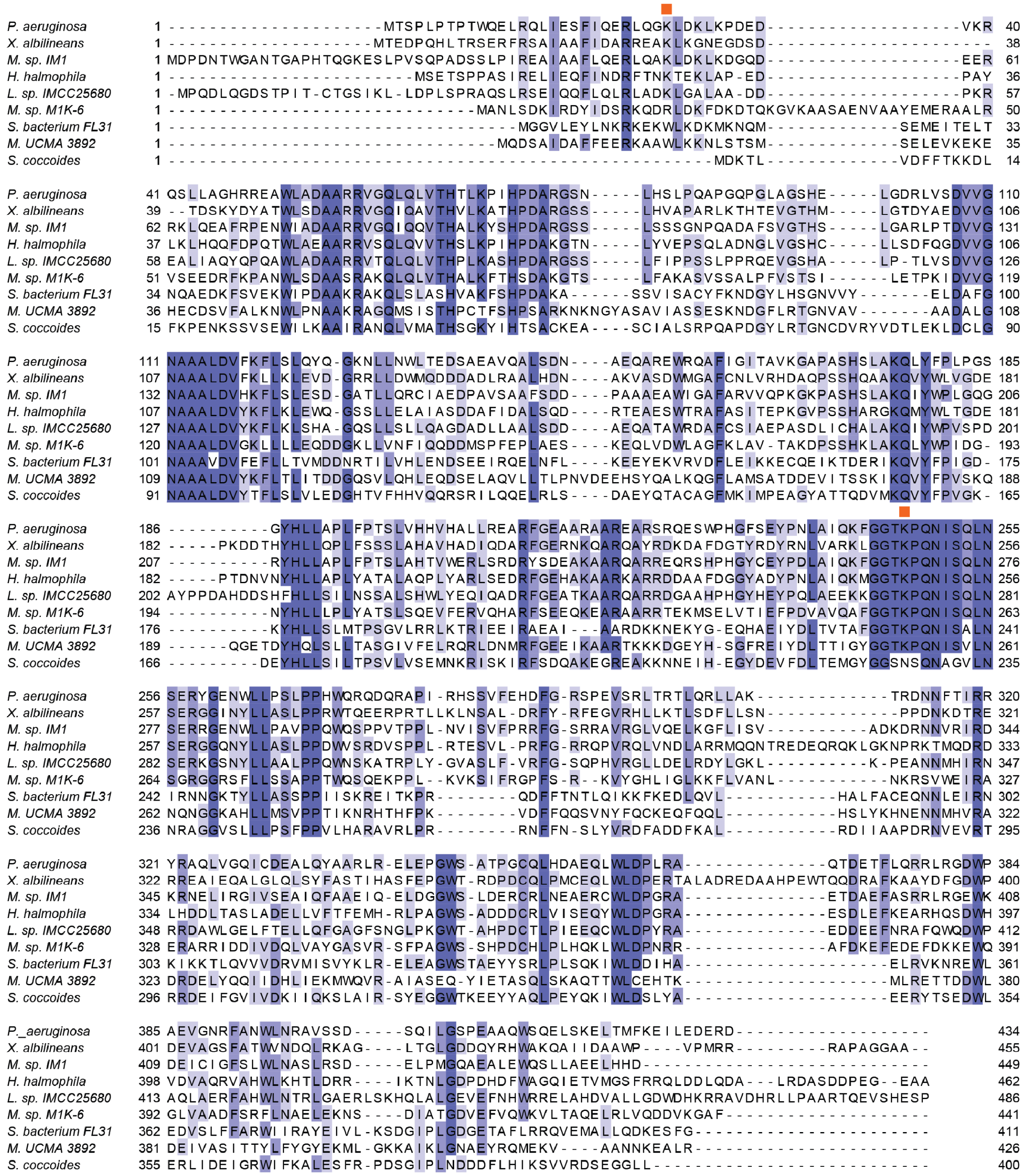

Figure S8. Electrostatic surface potentials for the interfaces of (**A**) AcrIF7:Cas8f (our model), (**B**) AcrIF2:Cas8f (PDB code 6B47), (**C**) AcrIF6:Cas8f (PDB code 6VQX), and (**D**) AcrIF10:Cas8f complexes (PDB code 6B48). In the *left* panel, AcrIF homologs are shown as cartoon diagrams, and Cas8f is shown as a surface representation color-coded by electrostatic surface potential. In the *right* panel, Acr homologs are shown as surface presentations, and Cas8f is shown as a cartoon diagram. AcrIF7 (*orange*), AcrIF2 (*red*), AcrIF6 (*purple*), AcrIF10 (*pink*), and Cas8f (*blue*) are presented in cartoon models according to the same color code as in Figure 5. Only the N-terminal region of Cas8f (residues 1–267) responsible for the interaction is shown for visual clarity, and Lys247 at the interface is shown as a surface-filling model. We note that the electrostatic surface potentials of Cas8f (*left* panel) were calculated based on the Cas8f coordinate of AcrIF6:Cas8f (PDB code 6VQX), because Cas8f in other structures does not harbor sufficient side chain atoms in the N-terminal regions to calculate surface potential using the Adaptive Poisson-Boltzmann Solver plugin of the Pymol program. Thus, the surface models of Cas8f are intended to visualize the interaction surface in a qualitative manner.

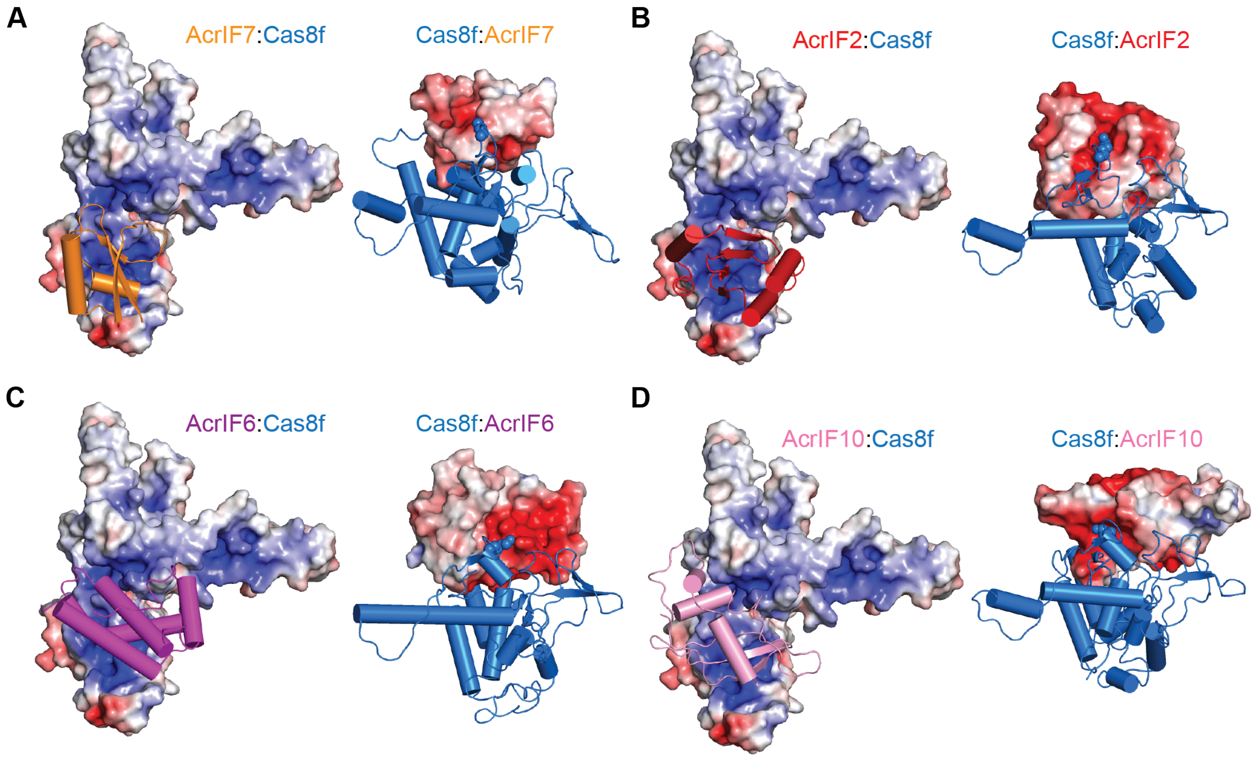

Figure S9. Uncropped gel images.

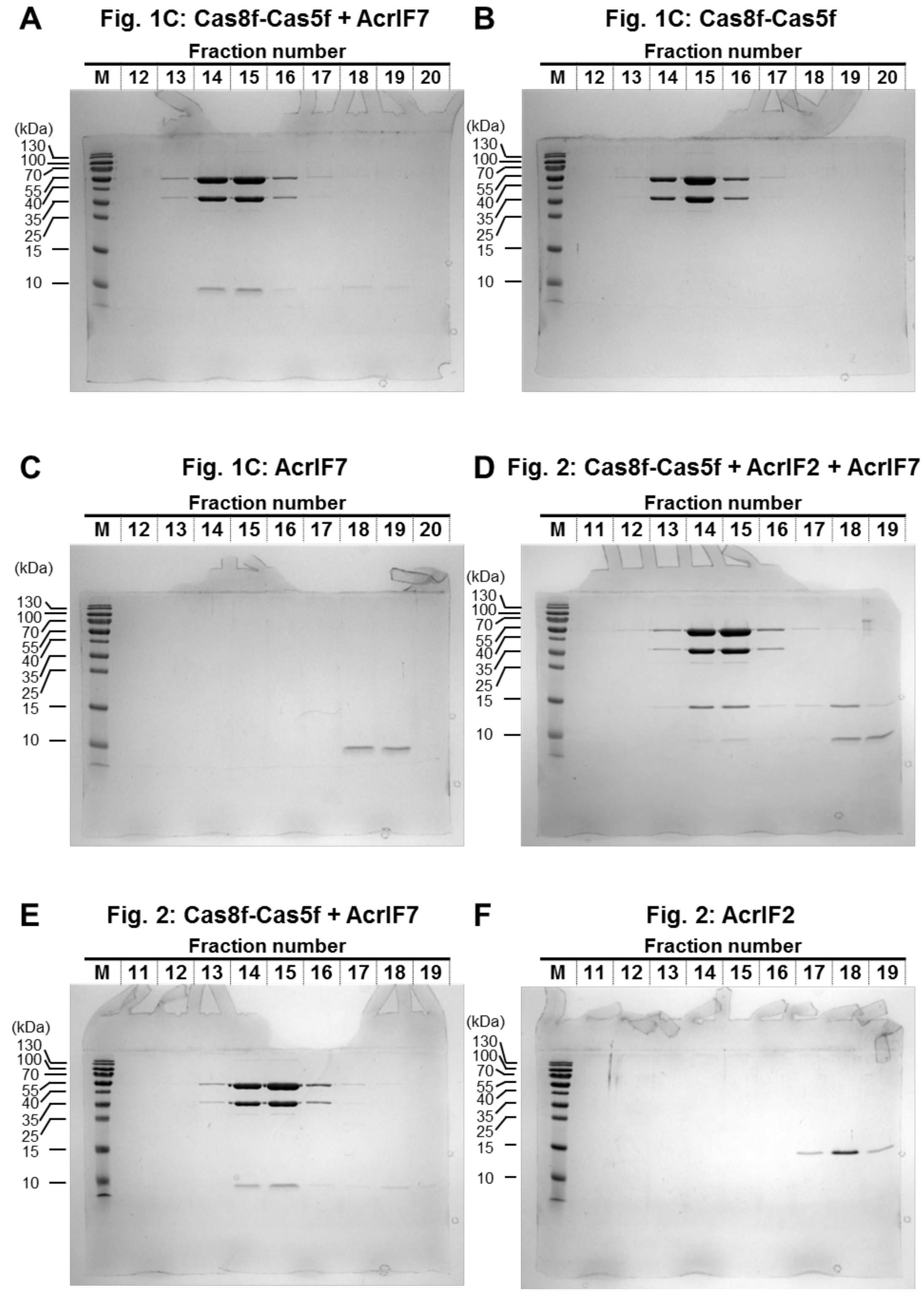

Supplementary References

1. Bondy-Denomy, J., Pawluk, A., Maxwell, K.L. and Davidson, A.R. (2013) Bacteriophage genes that inactivate the CRISPR/Cas bacterial immune system. *Nature*, **493**, 429-432.
2. Bondy-Denomy, J., Garcia, B., Strum, S., Du, M., Rollins, M.F., Hidalgo-Reyes, Y., Wiedenheft, B., Maxwell, K.L. and Davidson, A.R. (2015) Multiple mechanisms for CRISPR-Cas inhibition by anti-CRISPR proteins. *Nature*, **526**, 136-139.
3. Chowdhury, S., Carter, J., Rollins, M.F., Golden, S.M., Jackson, R.N., Hoffmann, C., Nosaka, L., Bondy-Denomy, J., Maxwell, K.L., Davidson, A.R. *et al.* (2017) Structure Reveals Mechanisms of Viral Suppressors that Intercept a CRISPR RNA-Guided Surveillance Complex. *Cell*, **169**, 47-57 e11.
4. Peng, R., Xu, Y., Zhu, T., Li, N., Qi, J., Chai, Y., Wu, M., Zhang, X., Shi, Y., Wang, P. *et al.* (2017) Alternate binding modes of anti-CRISPR viral suppressors AcrF1/2 to Csy surveillance complex revealed by cryo-EM structures. *Cell Res*, **27**, 853-864.
5. Guo, T.W., Bartesaghi, A., Yang, H., Falconieri, V., Rao, P., Merk, A., Eng, E.T., Raczkowski, A.M., Fox, T., Earl, L.A. *et al.* (2017) Cryo-EM Structures Reveal Mechanism and Inhibition of DNA Targeting by a CRISPR-Cas Surveillance Complex. *Cell*, **171**, 414-426 e412.
6. Hong, S., Ka, D., Yoon, S.J., Suh, N., Jeong, M., Suh, J.Y. and Bae, E. (2018) CRISPR RNA and anti-CRISPR protein binding to the Xanthomonas albilineans Csy1-Csy2 heterodimer in the type I-F CRISPR-Cas system. *J Biol Chem*, **293**, 2744-2754.
7. Wang, X., Yao, D., Xu, J.G., Li, A.R., Xu, J., Fu, P., Zhou, Y. and Zhu, Y. (2016) Structural basis of Cas3 inhibition by the bacteriophage protein AcrF3. *Nat Struct Mol Biol*, **23**, 868-870.
8. Pawluk, A., Staals, R.H., Taylor, C., Watson, B.N., Saha, S., Fineran, P.C., Maxwell, K.L. and Davidson, A.R. (2016) Inactivation of CRISPR-Cas systems by anti-CRISPR proteins in diverse bacterial species. *Nat Microbiol*, **1**, 16085.
9. Zhang, K., Wang, S., Li, S., Zhu, Y., Pintilie, G.D., Mou, T.C., Schmid, M.F., Huang, Z. and Chiu, W. (2020) Inhibition mechanisms of AcrF9, AcrF8, and AcrF6 against type I-F CRISPR-Cas complex revealed by cryo-EM. *Proc Natl Acad Sci U S A*, **117**, 7176-7182.
10. Hirschi, M., Lu, W.T., Santiago-Frangos, A., Wilkinson, R., Golden, S.M., Davidson, A.R., Lander, G.C. and Wiedenheft, B. (2020) AcrIF9 tethers non-sequence specific dsDNA to the CRISPR RNA-guided surveillance complex. *Nat Commun*, **11**, 2730.
11. Marino, N.D., Zhang, J.Y., Borges, A.L., Sousa, A.A., Leon, L.M., Rauch, B.J., Walton, R.T., Berry, J.D., Joung, J.K., Kleinstiver, B.P. *et al.* (2018) Discovery of widespread type I and type V CRISPR-Cas inhibitors. *Science*, **362**, 240-242.
